# Attenuation of wind intensities exacerbates anoxic conditions leading to sulfur plume development off the coast of Peru

**DOI:** 10.1371/journal.pone.0287914

**Published:** 2023-08-30

**Authors:** Edgart Flores, Ursula Mendoza, Cameron M. Callbeck, Rut Díaz, Arturo Aguirre-Velarde, Michael E. Böttcher, Lander Merma-Mora, Manuel Moreira, Maritza S. Saldarriaga, Emmanoel V. Silva-Filho, Ana L. Albuquerque, Matias Pizarro-Koch, Michelle Graco

**Affiliations:** 1 Programa de Maestría de Ciencias del Mar, Universidad Peruana Cayetano Heredia, Lima, Peru; 2 Millennium Institute of Oceanography, Universidad de Concepción, Concepción, Chile; 3 Department of Geological Sciences, Institute of Arctic and Alpine Research, University of Colorado, Boulder, Colorado, United States of America; 4 Dirección General de Investigaciones en Oceanografía y Cambio Climático, Instituto del Mar del Perú, Callao, Peru; 5 Facultad de Ciencias Veterinarias y Biológicas, Escuela de Biología Marina, Universidad Científica del Sur, Lima, Peru; 6 Department of Environmental Sciences, University of Basel, Basel, Switzerland; 7 Programa de Geoquímica, Universidade Federal Fluminense, Niterói, Rio de Janeiro, Brazil; 8 Dirección General de Investigaciones en Acuicultura, Instituto del Mar del Perú, Callao, Peru; 9 Geochemistry & Isotope Biogeochemistry Group, Department of Marine Geology, Leibniz Institute for Baltic Sea Research, Warnemünde, Germany; 10 Marine Geochemistry, University of Greifswald, Greifswald, Germany; 11 Interdisciplinary Faculty, University of Rostock, Rostock, Germany; 12 Dirección General de Investigaciones de Recursos Demersales y Litorales, Instituto del Mar del Perú, Callao, Peru; 13 Departamento de Geologia e Geofísica, Universidade Federal Fluminense, Niterói, Rio de Janeiro, Brazil; 14 Escuela de Ingeniería Civil Oceánica, Facultad de Ingeniería, Universidad de Valparaíso, Valparaíso, Chile; 15 Millennium Nucleus Understanding Past Coastal Upwelling Systems and Environmental Local and Lasting Impacts, Coquimbo, Chile; Naturhistoriska riksmuseet, SWEDEN

## Abstract

The release of vast quantities of sulfide from the sediment into the water column, known as a sulfidic event, has detrimental consequences on fish catches, including downstream effects on other linked element cycles. Despite being frequent occurrences in marine upwelling regions, our understanding of the factors that moderate sulfidic event formation and termination are still rudimentary. Here, we examined the biogeochemical and hydrodynamic conditions that underpinned the formation/termination of one of the largest sulfur plumes to be reported in the Peruvian upwelling zone. Consistent with previous research, we find that the sulfur-rich plume arose during the austral summer when anoxic conditions (i.e., oxygen and nitrate depletion) prevailed in waters overlying the upper shelf. Furthermore, the shelf sediments were organically charged and characterized by low iron-bound sulfur concentrations, further enabling the diffusion of benthic-generated sulfide into the water column. While these biogeochemical conditions provided a predicate to sulfidic event formation, we highlight that attenuations in local wind intensity served as an event trigger. Namely, interruptions in local wind speed constrained upwelling intensity, causing increased stratification over the upper shelf. Moreover, disturbances in local wind patterns likely placed additional constraints on wind-driven mesoscale eddy propagation, with feedback effects on coastal elemental sulfur plume (ESP) formation. We suggest ESP development occurs as a result of a complex interaction of biogeochemistry with regional hydrodynamics.

## Introduction

**Fig 1:**
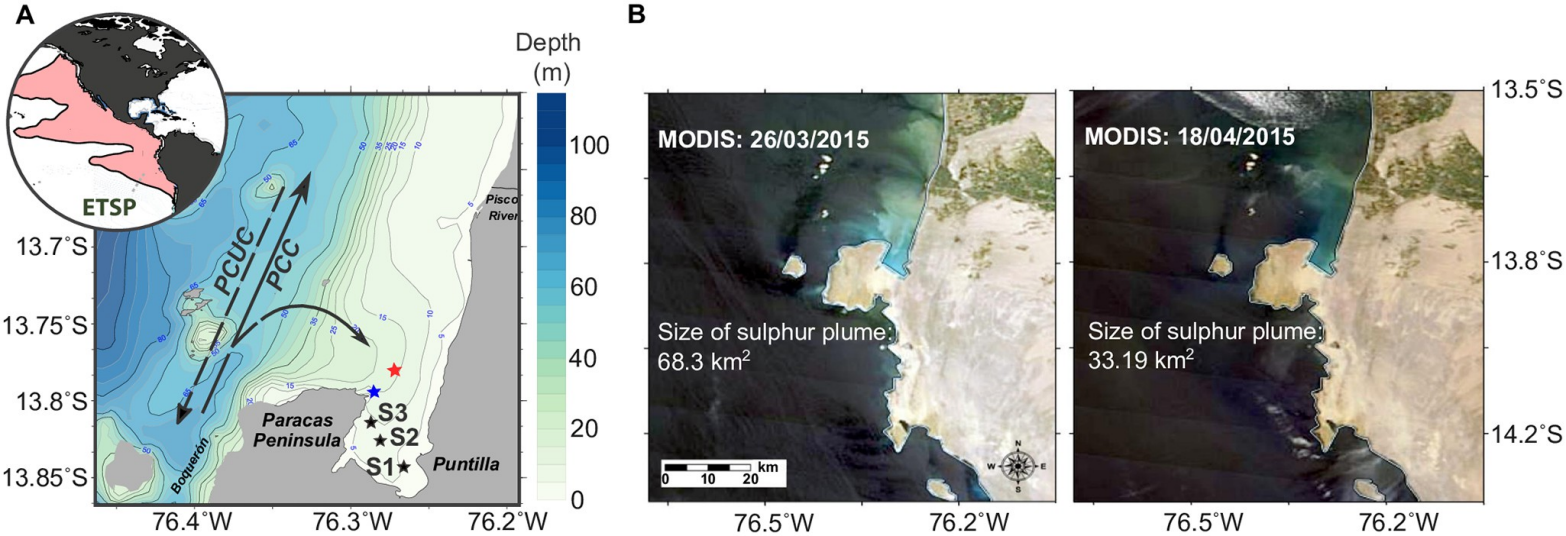
The location of stations sampled in the ETSP oxygen minimum zone and satellite images of elemental sulfur plumes. (A) Sediment core analyses and water column oxygen concentrations were performed at the stations indicated by the black stars (S1, S2, and S3). We measured the time series of bottom oxygen and the proxy of water column stratification near San Martin Harbor, which is indicated by the blue star. The red star denotes the position where wind satellite data was obtained by The Advanced Scatterometer. The main currents discussed in this study include the surface Peru Coastal Current (PCC) and the subsurface Peru-Chile Undercurrent (PCUC), with the flow direction indicated by the dashed and solid arrows, respectively. The world atlas globe (as shown in Callbeck et al., 2021), indicates the extent of the oxygen minimum zone, with the contour line representing 20 μM of O_2_. The entire area of the oxygen minimum zone is denoted by the red shading. The abbreviation stands for the Eastern Tropical South Pacific (ETSP). (B) Satellite images of elemental sulfur plumes detected by MERIS (true-color) on 26^th^ March 2015, and on 18^th^ April 2015 in Paracas Bay.

Oxygen minimum zones (OMZs) are areas in the ocean where high primary productivity combined with poor regional ventilation contribute to exceptionally low dissolved oxygen concentrations in the water column (< 20 μmol kg^-1^). The oxygen-depleted waters, including the underlying sediments of the OMZs, are known for harboring a highly active sulfur cycle [1–5]. In extreme cases, overactive benthic sulfide production can result in the periodic release of vast quantities of hydrogen sulfide (H_2_S), that exceed a range of 2–3 mol m^-2^ d^-1^ [6, 7] into the overlying water column, a condition known as a hydrogen sulfide/sulfur plume [8]. The subsequent oxidation of H_2_S generates elemental sulfur as a by-product, which can be detected in the water column by satellite remote sensing as a milky turquoise discoloration (e.g. Fig 1). Hereafter, we refer to a sulfidic event as an elemental sulfur plume (ESP) [5]. To date, most of the major OMZs located off the coast of Peru, Chile, India, and Namibia have reported ESPs in shallow coastal waters (< 200 m; [5, 8–14]. These events are of growing concern given that micromolar levels of sulfide can be toxic to higher life, with potential downstream implications for regional fisheries and human health [15–19]. Moreover, from a biogeochemical perspective, the linkage of sulfur cycling with other coupled element cycles (such as nitrogen) has great potential to accelerate regional nitrogen loss and the emission of climate-relevant gases such as N_2_O [2].

ESPs arise due to a tight coupling of benthic and pelagic microbial activity. The sulfide that accumulates in the OMZ water column is generated in the underlying sediments as a result of dissimilatory sulfate reduction, driven by the activity of sulfate-reducing bacteria [20–25]. Sulfate reducers, which are dependent on organic matter for energy and growth, are fueled by the elevated rates of organic matter loading to the sediment, which are commonly associated with productive marine upwelling regions. Produced benthic sulfide that escapes abiotic precipitation in the sediments by reacting with amorphous iron (i.e., Acid Volatile Sulfides-AVS, chromium reducible sulfur-CRS, or pyrite-FeS_2_) [7, 26, 27], can eventually diffuse into the overlying water column. Here, the benthic sulfide flux is oxidized by pelagic sulfide-oxidizing bacteria. These bacteria, typically oxidize H_2_S to SO_4_^2-^, or to other intermediate sulfur species, such as elemental sulfur (S^0^), thiosulfate (S_2_O_3_), and/or sulfite (SO_3_^2-^), using mostly nitrate as the electron acceptor (given the general absence of dissolved oxygen) [5, 8, 13, 28]. Due to the activity of sulfide-oxidizing nitrate-reducing bacteria, ESPs can be further evidenced by the presence of nitrate-depleted bottom waters and the accumulation of high concentrations of elemental sulfur (> 6 μM; [13]). A fortunate property of elemental sulfur is its reflectance at an optimal wavelength, which enables the detection of ESPs by satellite remote sensing [29, 30]. Both the remote sensing of sulfur plumes and shipboard measurements of dissolved sulfide have shown that ESPs originate over the upper shelf (< 200 m), where coastal upwelling drives high primary productivity [5, 9, 29–31]. Moving into the offshore OMZ (> 200 m) and away from the benthic sulfide flux [32], lowers the potential for ESP formation; however, coastal sulfur plumes may still extend into the open ocean, as a consequence of cross-shelf transport [13].

New evidence has revealed that ESPs are not as scarce as previously believed in OMZs [2]. ESPs in OMZs are not as recurrent features in the austral summer-autumn seasons when upper shelf anoxia prevails (i.e., when bottom waters are depleted in both oxygen and nitrate). This window of anoxia can last between 180–200 days and generally coincides with high primary productivity in surface waters that, in turn, manifests as high rates of organic matter loading to sediments [29, 33]. Modeling studies have estimated that the release of benthic sulfide is triggered when organic matter deposition rates to the sediments exceed 20 mmol m^−2^ d^−1^ [34]. Hence, the austral summer-autumn season is tightly linked with ESP formation, with exceptions occurring during El Niño events, which tend to hinder ESP formation due to the introduction of an oxygenated water mass over the shelf [29]. Other prerequisites to ESP formation include the development of stagnant bottom waters over the shelf, which facilitates greater water column stratification, and hence, a drawdown of water column oxygen and nitrate concentrations. For example, off the coast of India, heavy rainfall events that result in a fresh/warm water lens developing overlying saline/cold upwelled waters, have been shown to isolate the bottom waters from the coastal upwelling, thereby driving water column stratification and ESP formation [19, 35]. Similarly, ESPs have been reported over the upper shelf of Peru (12°S) during near-stagnant circulation [32].

Despite some consistent patterns regarding ESP formation (i.e., anoxia, stagnant/stratified waters, organic matter loading), our ability to predict where/when ESPs occur in OMZs during the austral summer-autumn season is still limited. For example, ESPs can fluctuate in intensity over a period of days to weeks within the austral summer-autumn period, hinting at possibly other small-scale hydrodynamic features that moderate their development and termination [5, 13, 28, 30]. Mesoscale eddies, for example, have been implicated as ostensible contributors to ESP formation. Mesoscale eddies, roughly 90 km in diameter, arise from instabilities in the vertical shear of coastal wind-driven currents, which typically develop along topographical bends (i.e., from protruding peninsulas). An oceanographic survey that tracked a developing mesoscale eddy off the coast of Peru, showed that as the eddy developed in lower shelf waters, current velocities associated with the upper shelf had decreased significantly [36]. Interestingly, the main shelf undercurrent, which normally propagates along the shelf, was temporarily diverted around the eddy creating a so-called “shadow zone”. During this period, the enhanced water column stratification in the “shadow zone” lasted upwards of two weeks, providing sufficient time to draw down available nitrate in upper shelf bottom waters [36]. An accompanying ESP later developed in the same waters adjacent to the mesoscale eddy [13], leading to the hypothesis that eddies may be linked, at least in part, to ESP formation and termination [2]. Furthermore, in the Namibian upwelling region, ESPs share a close link with local wind intensities, whereby larger ESP events are correlated with lower wind intensities [33]. How local wind-forcing and regional hydrodynamics impact ESP development in the Eastern Tropical South Pacific (ETSP) region–one of the most productive coastal upwelling regions in the world–remains unclear.

To this end, we examined ESP development during the austral summer-autumn period in the ETSP OMZ, and specifically, off the coast of Peru between 13°–15°S–an area that is prone to ESP formation [8, 13, 29]. The waters off the coast of Peru are permanently depleted of dissolved O_2_ concentrations (<10 nM) [37], with nitrate in bottom waters being the last barrier to ESP formation [8, 13]. The recurrence of ESPs in this region is attributable to high surface primary productivity coupled with the elevated rates of organic matter loading to upper shelf sediments [38]. The high surface primary productivity is sustained by upwelling-favorable winds, which are strongest in austral winter/spring [29, 39]. Critical to upper shelf primary productivity and shelf biogeochemistry are the Peru Coastal Current (PCC) and the Peru-Chile Undercurrent (PCUC). The PCC surface current flows in the direction of the equator (Ekman surface layer), while the PCUC is a subsurface current that flows poleward at velocities of up to 10–15 cm s^-1^ [40]. Instabilities in the vertical shear of the PCC and PCUC, especially near topographical bends (e.g., the Paracas Peninsula), commonly lead to mesoscale eddy development over the lower shelf between 13°– 15°S [36, 40–42].

Our study employs satellite remote sensing to investigate how changes in the hydrodynamic conditions, such as interruptions in the local wind, changes in bottom water temperature, and eddy propagation, influence the development of ESPs. We combine our remote sensing analyses with a characterization of shelf biogeochemistry to provide a snapshot of conditions that prevailed during the austral summer-autumn ESP period. Specifically, we collected samples for water column nutrient (oxygen and nitrate) analysis, as well as sediment cores in Paracas Bay (13°S; Fig 1A), to examine pore water sulfur species, sedimentary organic matter, Fe sulfide contents, and calculate hydrogen sulfide fluxes across the sediment-water interface. We find that interruptions in the local wind were key triggers of ETSP-ESP formation. Wherein, we propose that attenuations in local wind not only constrain upwelling intensity inducing water column stratification but also impede mesoscale eddy propagation, altogether enhancing coastal ESP development.

## Materials and methods

### Satellite remote sensing

**Fig 2:**
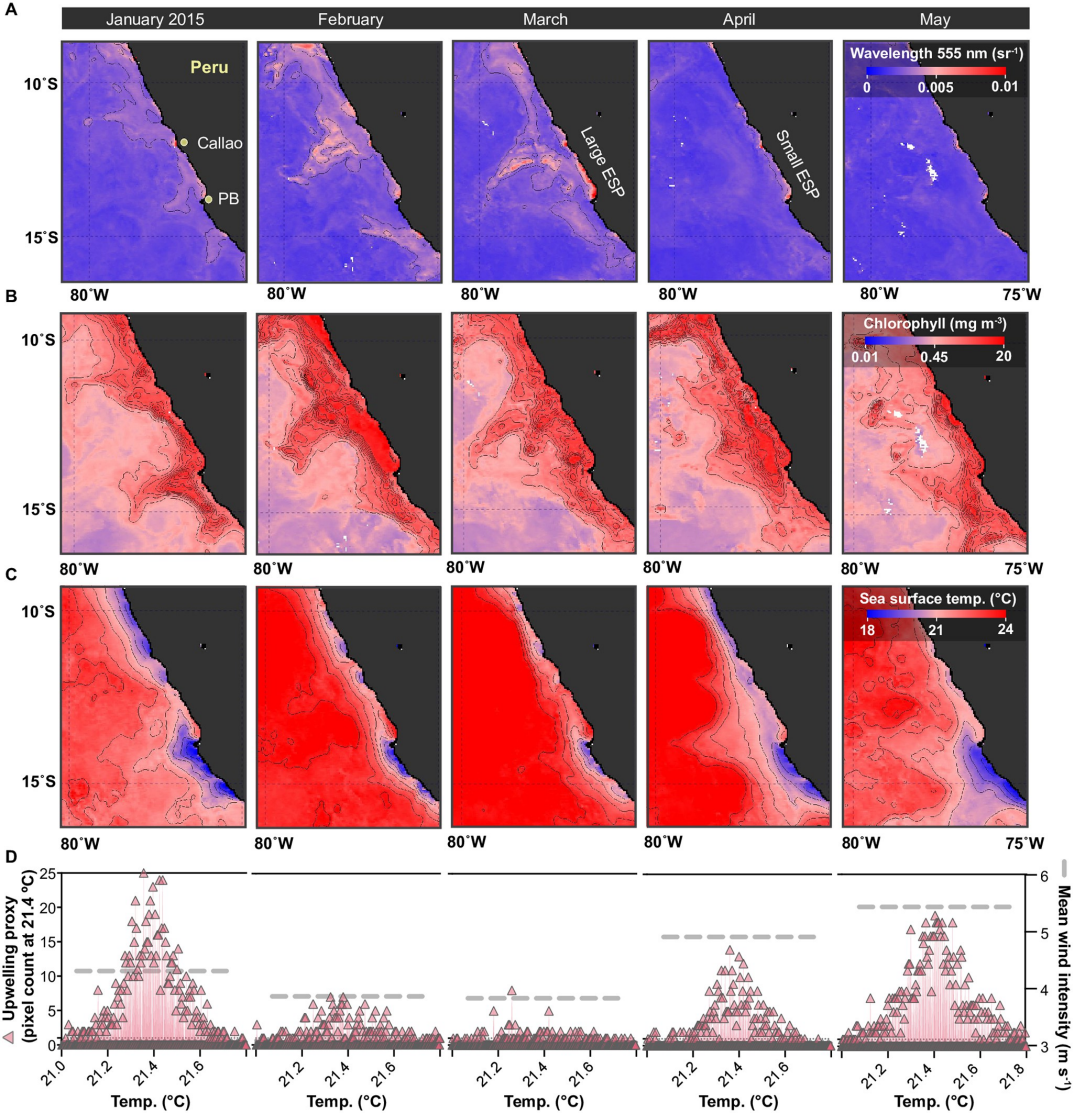
Distribution and intensity of ETSP sulfur plumes relative to other remotely detected oceanographic conditions. The monthly composite satellite images were taken off the coast Peru during the austral summer-autumn period in 2015. (A) MODIS images of the elemental sulfur plume as detected by the water reflectance wavelength at 555 nm (optimal band for ESP detection). (B) MODIS images of near-surface chlorophyll concentrations (expressed in relative units), and (C) sea surface temperature. (D) Pixel histogram plots of sea surface temperature data. The upwelling intensity is indicated at temperatures of around 21.4°C. Moreover, this plot is overlaid with a grey dotted line denoting the mean monthly wind intensities. The mean wind intensity values were determined from the daily values presented in Fig 7.

We identified sulfur plumes according to the water-leaving reflectance at 488 nm (representing the true color image; [43], and with 555 nm identified as the optimal band for So detection [44]) (Figs 1B and 2A). The images were acquired by MODIS (Moderate Resolution Imaging Spectroradiometer) on NASA’s Terra platforms and images were processed using SeaDas software (www.seadas.gsfc.nasa.gov/). We estimated the size of the sulfur plume in March 2015 according to the pixel histograms collected for the water reflectance wavelength at 555 nm (S1 Fig). Calculating the size of the sulfur plume is complicated, as such plumes often develop on the upper shelf but can be later transported into the open ocean due to cross-shelf advection [13]. To encompass the full scope of the plume (i.e., both offshore and coastal sulfur-containing pixels), we performed a background excess calculation, in which the binned pixels (at 555 nm) associated with a non-ESP month (May) were subtracted from the binned sulfur-containing pixels of the March 2015 period (ESP scenario). Given the known area of a pixel, we deduced that the March ESP reached an estimated size of 16,963 km^2^.

Daily surface wind data, with a spatial resolution of 0.25° in longitude and latitude (25 km), were acquired by Advanced Scatterometer (ASCAT) aboard Metop-A and Metop-B satellites. Note that the wind component time series, in both the cross-shore (west-east; U) and the alongshore (south-north; V) winds, were extracted at 13°46’ S, 76°16’ W (red star in Fig 1A), from January 1^st^ to 30^th^ June 2015. Sea surface temperature and chlorophyll were downloaded from MODIS; sea surface height altimetry was acquired by AVISO. Images were further processed using SeaDas software as mentioned above.

### Water column nitrate, oxygen, and temperature measurements

**Fig 3:**
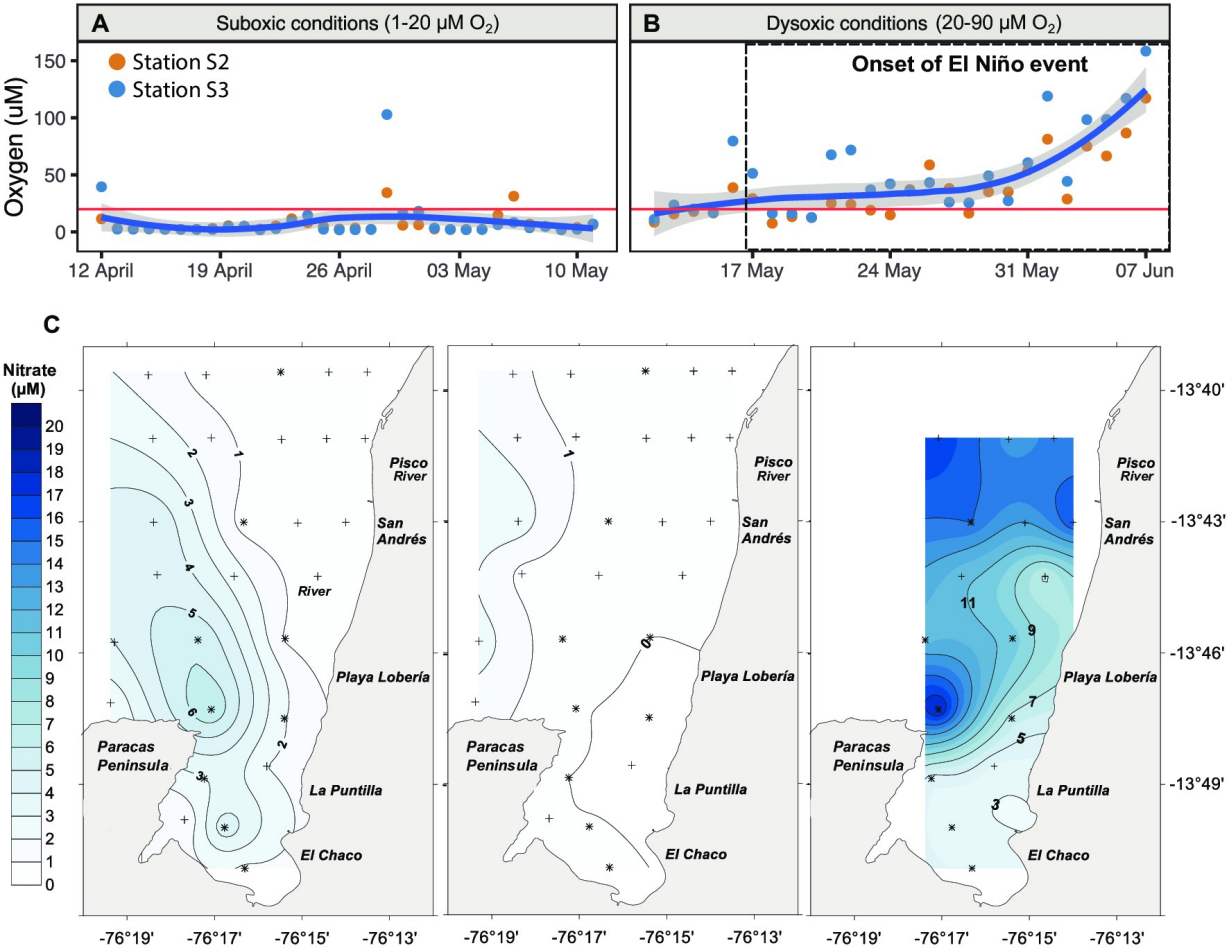
Dissolved bottom water oxygen and nitrate concentrations off the coast of Peru. Synoptic time series images were taken during the austral summer-autumn period in 2015. Shown are (A) bottom water oxygen concentrations sampled in April to mid-May, and (B) in mid-April to early June 2015. In both (A, B), the blue line denotes the smoothed daily mean, while the grey shading illustrates the confidence interval. The red line indicates the dissolved oxygen threshold for suboxic (< 20 μM) and dysoxic (20–90 μM) conditions, as previously defined according to Wright [42]. Note that oxygen concentrations for station S1 are shown in S3 Fig. (C) Bottom water nitrate concentration (μM) from left to right: November 2014, March 2015, and July 2015, respectively, adapted from Merma [46].

The fieldwork was carried out along a three-station transect: station S1 (13°50’35.88” S, 76°15’59.46” W), station S2 (13°49’32.16” S, 76°16’53.1” W), and station S3 (13°48’52.26” S, 76°17’14.16” W) (S1 Table, Fig 1A), from 12^th^ April to 7^th^ June 2015. At each station, an autonomous MiniDO_2_T data logger was deployed 20 cm above the sediments to record the dissolved oxygen of the sediment-overlying bottom water (Fig 3A and 3B). Here, we define dissolved oxygen concentration cutoffs according to Wright et al. [45]: anoxic (< 1 μM O_2_), suboxic (1–20 μM O_2_), and dysoxic (20–90 μM O_2_). In addition, to provide context for the summer-autumn 2015 fluctuations to the climatological average, we used daily records of surface and bottom water temperature measured between 2006 and 2015 in a nearshore station (ca. 6m depth), next to the San Martin Harbor, by the environmental monitoring program of the PLUSPETROL Company [46] (Fig 7C). The difference between surface and bottom water temperature (ΔT °C) has been previously used as a proxy of water stratification [47] (Fig 7D).

After the completion of the "Borde Costero" Project 2014–2015, nitrate concentrations in thawed samples were determined using the Griess–Ilosvay method [48] via spectrophotometry at IMARPE. The analytical precision was ±0.5 μM (Fig 3C, for more detail see Merma-Mora, [46]).

### Sediment analysis

We collected sediment for the elemental analysis of carbon-nitrogen-sulfur (CNS), inorganic sulfur species, and sulfide-sulfate-chloride in pore water. We obtained two sediment cores from our study sites by diving and manually placing acrylic tubes (Ø = 6 cm, 50 cm length) into the sediment. At stations S1-S3, sampling was performed weekly from 12^th^ April to 7^th^ June 2015. Recovered cores were subsequently refrigerated at ~4°C and processed within 2 hours. The sediment pore water, as well as the overlying waters, were extracted using rhizons [49] from one of the replicate cores via the pre-drilled holes. The pre-drilled holes used for subsampling the overlying water were situated ~5 cm above the sediment-water interface; while the pore water was extracted from 0–5 cm (1 cm intervals), from 5–12 cm (2 cm intervals), and 12–20 cm (4 cm intervals).

To prevent the oxidation of dissolved pore water HS^-^, 1 mL subsamples were immediately fixed in 100 μL of Zn-acetate solution (5% w/v), and then stored at 4°C. Additionally, the subsampling was done inside a nitrogen-flushed glove bag. The dissolved sulfide was analyzed, according to the diamine method, with a detection limit of 1 μM. For the analysis of SO_4_^2-^ and chloride (Cl^-^) concentrations, we aliquoted 20 μL of sample into 1 mL of milli-Q water, which was then measured by ion chromatography on a Metrohm 850 [50], with a detection limit of 0.1 μM. In this study, we used the Cl^-^ as an indicator of changing pore water salinity. The accuracy of the analysis was greater than 5%, which was evaluated by the analysis of certified reference materials (Fluka^®^) and tracked by the National Institute of Standards and Technology, and the relative error was 3.2%.

We analyzed the solid phases of sulfur monthly at the beginning and end of the sampling period. The sediment cores were extruded and sliced into 1 cm thick sections, at the same depths where pore water samples were obtained. The recovered sediment fractions were immediately fixed in Zn-acetate (20% w/v), with work taking place inside a nitrogen-flushed glove bag. Fixed samples were stored at -20°C for later analyses of acid volatile sulfides (AVS: FeS + minor ƩH_2_S) and chromium reducible sulfur (CRS: FeS_2_ + minor S^0^).

AVS and CRS concentrations were determined from 1 g of wet sediment involving a two-step distillation process with cold 6M HCl followed by heating (80°C) with 2M acidic CrCl_2_ solution [51]. The liberated H_2_S was collected in Zn-acetate traps, and its concentration was determined with a UV–Vis spectrophotometer Nova 2000 series as per Cline [52]. The overall precision for duplicate distillation was ±10 and ±15% for the AVS and CRS collected fractions, respectively. The percent sulfur content was calculated relative to the dry fraction of the sediment.

We also determined the porosity by calculating the difference between the wet weight and the dry weight of the sediments, as well as the total organic carbon (TOC) and total sulfur (TS) content from separate sediment fractions. To quantify TOC, we weighed duplicate subsamples into silver cups and decarbonated them by adding 200 μL of 1N HCl, which was then incubated at 40°C for 24 hours [53]. After desiccation, the cups were closed and analyzed for total carbon (TC), total nitrogen (TN), and total sulfur (TS) contents using a Flash 2000 elemental analyzer (Thermo Scientific) [54]. The measurements were expressed as micromoles per gram of sediment (μmol g^-1^) and were presented as their equivalent weight percentage of dried sediment (wt.%). Total inorganic carbon (TIC) contents were calculated from the difference between TC and TOC measurements. The average analytical uncertainty, based on duplicate sediment sample analyses, was 2.8 wt. % for TOC and 2.6 wt. % for CNS.

### Flux calculation

Fick´s first law of diffusion under steady-state conditions was used to calculate the diffusive fluxes across the benthic boundary using concentration gradients between the uppermost pore water sample (0.5 cm) and the sediment-overlying bottom water according to Berner and Berner [55]:

$$J=-\varphi \;{\text{D}}_{sed}\frac{\partial C}{\partial z}$$ (1)

where *J* is the diffusive flux (mmol m^-2^ d^-1^), *φ* is the porosity, *D*_*sed*_ is the sediment diffusion coefficient (m^2^ d^-1^), *∂C* denotes the concentration of sulfide (mmol/m^3^) and *∂z* is the depth (m).

The diffusion coefficients (m^2^ d^-1^) for sulfide in seawater (*D*_*sw*_) were adjusted to *in situ* temperature according to Boudreau [56], and Yuan-Hui and Gregory [57] using the Stokes-Einstein equation. Diffusion coefficients for sediments (D_*sed*_) were calculated as:

$${\text{D}}_{sed}={\text{D}}_{sw}/\theta^{2}$$ (2)

and, tortuosity (θ) was derived from porosity using the following relationship from Boudreau et al. [56]:

$$\theta^{2}=1-\text{ln}\;(\phi^{2})$$ (3)

where ϕ is the sedimentary porosity (m^3^ pore water m^-3^ bulk sediment). Negative values represent a flux from the water column into the sediment while a positive value signifies a flux from the sediment into the water column. The advection, bioturbation, and other processes were not calculated. The diffusive flux calculations are shown in the S4 Table.

### Statistical methods

For statistical analysis, as a criterion for determining the stationarity in the time series, the Dickey-Fuller Test (*p* < 0.05) was employed. The cross-correlation function (CCF) for each time step (lag, measured in days) was used to compare the surface temperature, bottom temperature, ΔT, and wind speed time series at 95% confidence. The summary of significant CCF correlations is listed in the S5 Table.

Daily averages on *in situ* measurements of temperature (ºC) and oxygen (μM) were calculated from the time series and were smoothed using a locally weighted scatterplot smoothing (LOESS) curve for each sampling station. Principal Component Analysis (PCA) was performed on the geochemical data, previously normalized, to explore and compare the grouping of variables at the beginning (April) and the end (June) of the sampling campaign. Furthermore, we analyzed the grouping of the three stations (S1, S2, & S3) comparing each depth (1, 2, 3, 5, 7, 9, 11, 15, & 20 cm) and its spatial distribution of the sampling sites. In the PCA, the vector length reflects the variability associated with the single variable, and the cosine of the angle between vectors reflects the degree of correlation between variables [58], the significant level was set at p < 0.05. All data treatment and statistical analyses were performed using the Factoextra package in R [59].

## Results

### ESP detection and the biogeochemistry of ETSP waters

From the remote detection of sulfur plumes, we identified prominent ESPs from both ocean true color images and by the elemental sulfur reflectance wavelength during the austral summer period in 2015 (Figs 1B and 2A). Specifically, we observed both small and large ESPs occurring from January to April, with the events mostly dissipating in May (Fig 2A). The largest and most intense ESP occurred in March 2015, which encompassed an area of approximately 17,000 km^2^ (entails both the coastal and offshore ESP, see Methods for details). The ESP detected in March was most intense in coastal waters between Callao and Paracas Bay (greatest water reflectance values), but remnants of the sulfur plume were visible in offshore filaments that extended as much as 270 km from the coast.

Our analysis in the Pisco area during austral summer/autumn, consistent with previous studies [60, 61], revealed elevated primary productivity (Fig 2B). Specifically, we observed high primary productivity in the coastal shelf waters, which is driven, in large part, by the PCUC poleward undercurrent that introduces nutrients to the euphotic zone. Analogous to the detection of offshore sulfur plumes, primary productivity also extended offshore in the form of discrete filaments. Along the coast, the presence of the PCUC upwelling was evidenced by the narrow band of cool waters (mostly at 21.4°C), which was prominent in January across a large swath of the coast, except for the area extending from Callao to Pisco (Fig 2C). Due to the Callao-Pisco topographical bend, this partly sheltered region tends to experience limited/moderate contact with the PCUC, explaining the warmer surface water temperatures relative to coastal areas situated further South. It should be noted, that due to the shallow nature of Paracas Bay (~12 m depth), the PCUC is largely negligible as it mainly flows between 100–150 m water depth [40]; instead, these waters are in close contact with the PCC (surface current). A cursory inspection of our aerial images reveals that ESP development is especially prone in the sheltered Callao-Pisco topographical bend, where upwelling is present, but more limited (Fig 2A and 2D). In March, our tracked ESP, emerged when both upwelling and the mean monthly wind intensities (in both the alongshore and cross-shore directions; S2 Fig) were weak, particularly, in contrast to other months, such as in January and April (Fig 2D).

In situ measurements of dissolved water column oxygen and nitrate, at the S2 and S3 monitoring stations, affirm that the shallow coastal waters, in, and around, Paracas Bay were conducive to ESP formation in the austral summer of 2015. From April 12 to May 10, 2015 water column dissolved oxygen concentrations were mostly low (< 20 μM) or depleted (< 1 μM; Fig 3A and 3B). While dissolved oxygen data is not available before April (i.e., during the ESP in March), previous studies have consistently shown that the ETSP summer is typically associated with low/near zero O_2_ concentrations in upper shelf bottom waters from November to April [8, 13]. Similarly, we find relatively low bottom water nitrate concentrations over the upper shelf during the March ESP event (Fig 3C). Specifically, from November 2014 to July 2015 nitrate concentrations over the upper shelf ranged from 0–11 μM; however, concentrations were at their lowest in March 2015 (0–1 μM), when compared to November 2014 (1–5 μM) and July 2015 (3–11 μM). The higher nitrate and oxygen concentrations observed from mid-May onward were the result of an El Niño event (Fig 3A–3C), which introduced an oxygenated and nitrate-rich water mass over the upper shelf. ESPs were largely absent following the onset of the El Niño period (Fig 2A).

### Benthic sulfide and its flux into shallow waters

**Fig 4:**
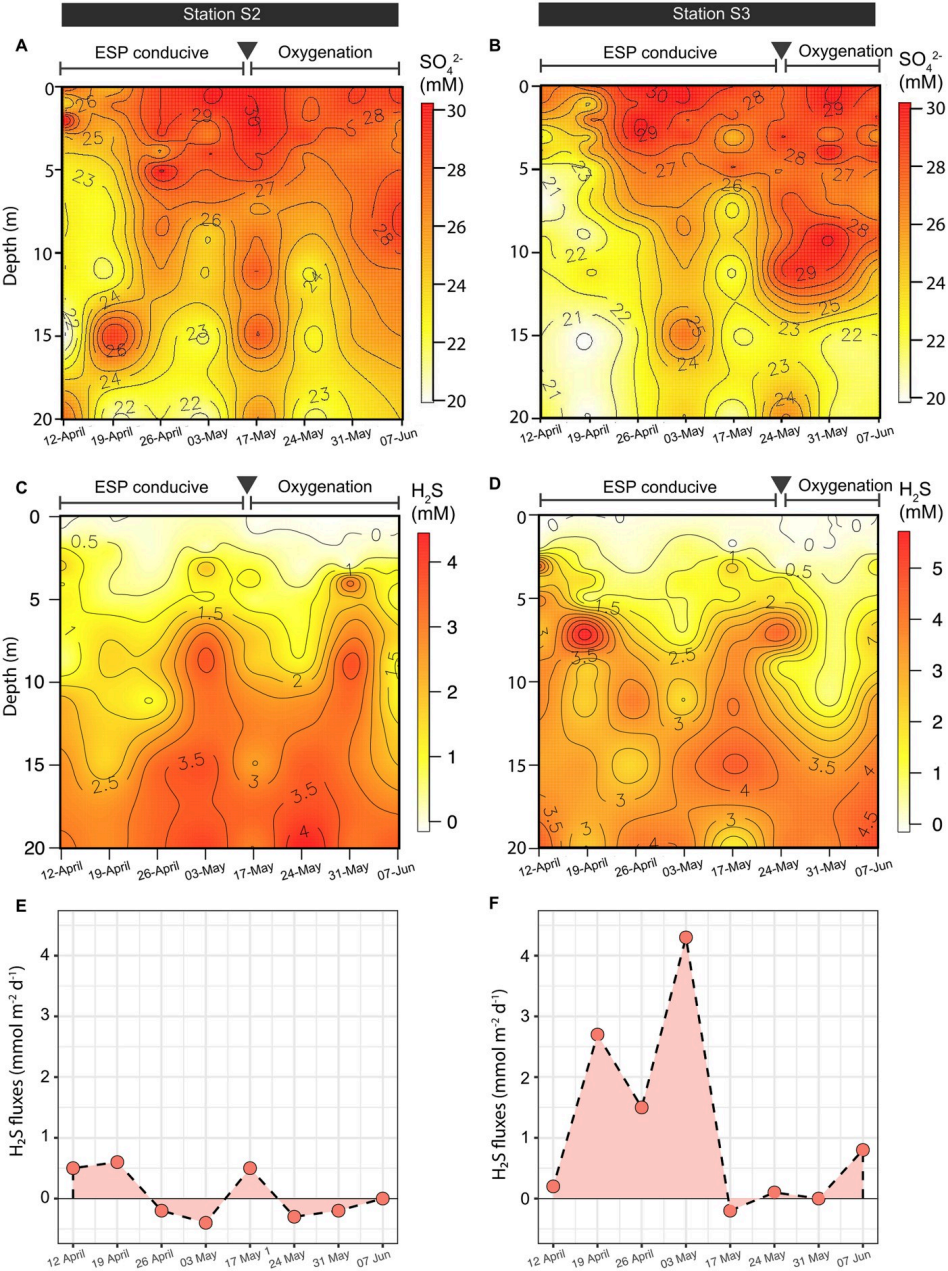
Vertical and temporal distribution of pore water sulfate and sulfide concentrations from sediment cores recovered in Paracas Bay. (A-D) Pore water SO_4_^2-^ and H_2_S concentrations are from cores taken at stations S2 and S3 (see Fig 1A). The inverted grey triangle denotes the shift from anoxic (oxygen and nitrate depletion) to oxygen-rich conditions introduced by the El Nino event, according to water column values presented in Fig 3. (E, F) Estimated benthic sulfide fluxes (mmol m^-2^ d^-1^) at stations S2 and S3, respectively. Additional vertical profiles of sulfate and sulfide can be found in S4 Fig.

Porewater analysis of sulfide and sulfate concentrations from cores recovered at stations S2 and S3 (in Paracas Bay) showed patterns consistent with previous ETSP sediment studies [47, 62]. From April to June, the vertical profiles (down to 20 cm) of pore water H_2_S (0–5 mM) and SO_4_^2-^ (20–29 mM) exhibited a wide range of concentrations (Fig 4A–4D; S4A and S4B Fig, S2 Table). The Cl^-^/SO_4_^2-^ molar ratio remained relatively stable, ranging from 0.10 to 0.18 (S4C Fig, S2 Table). As expected, pore water profiles of H_2_S showed an increase with sediment depth and were inversely correlated with SO_4_^2-^ concentrations (Spearman *ρ* = -0.73, *p-value* < 0.01). The lowest H_2_S concentrations (< 0.03 mM) were observed in the surface sediments (<5 cm), and conversely higher H_2_S concentrations were reported in the deeper sediments (7–20 cm).

Porewater analysis of sulfide and sulfate concentrations from cores recovered at stations S2 and S3 showed weekly variations between April and June in both the accumulative inventories of the analytes and their penetration depth. From April 12^th^ to June 7^th^, the base of the sulfate chemocline (demarcated at 27 μM) gradually extended deeper into the sediment, shifting from 5 cm in April to 10 cm in June (Fig 4A and 4B). The sulfide chemocline also deepened at station S3 over the same period; however, this trend appeared less clear at station S2 (Fig 4C and 4D). Moreover, in June following the oxygenation event (El Niño), sulfide concentrations were reaching nearly zero micromolar in the upper sediment layer at both stations S2 and S3. Overall, the transition from April to June, observed a drop in the cumulative sulfide pool of 29% and 13% at stations S3 and S2, respectively (Table 1). Based on the measured sulfide gradient, the estimated benthic sulfide flux ranged from -0.4 to 4.3 mmol m^-2^ d^-1^ between April and June (Fig 4E and 4F). April was mostly associated with a positive benthic sulfide flux at both stations S2 and S3, indicating that sulfide diffused into the water column from the sediments. Whereas cores analyzed in June were mostly associated with a so-called “sulfide deficit” in the uppermost sediment layers [2], which is diagnostic of sulfide removal, likely coupled to its oxidation with nitrate and/or oxygen introduced via El Niño.

**Table 1:** Sediment biogeochemistry data obtained from cores retrieved at stations S1, S2, and S3 (see Fig 1A) during the austral summer-autumn period in 2015. The range, standard deviations (±), and mean were calculated from sediment intervals values collected from 0–20 cm. It is important to note that sediment cores were analyzed under two contrasting biogeochemical conditions: anoxic (April, pre-El Niño) and oxic (July, post El Niño) conditions. The abbreviations used in the table correspond to the following parameters: TIC (total inorganic carbon), TC (total carbon) TN (total nitrogen), TS (total sulfur), AVS (Acid Volatile Sulfides), CRS (chromium reducible sulfur).

Parameter	April			June		
	Station 1 average (min/max±sd)	Station 2 average (min/max±sd)	Station 3 average (min/max±sd)	Station 1 average (min/max±sd)	Station 2 average (min/max±sd)	Station 3 average (min/max±sd)
TIC (%)	2.90 (2.34 /3.46±0.31)	2.45 (1.81 /2.69±0.27)	2.84 (0.36 /3.93±0.96)	2.43 (1.10 /2.84±0.52)	2.49 (1.32/3.15±0.47)	3.01 (1.74/3.60±0.53)
TC (%)	3.95 (3.30/4.57 ±0.43)	3.60 (2.79/4.31 ±0.53)	4.72 (4.17/5.47 ±0.96)	3.65 (3.32/4.33 ±0.34)	3.65 (3.05/4.42 ±0.51)	4.31 (3.26/5.35 ±0.61)
TN (%)	0.53 (0.39/0.65 ±0.09)	0.44 (0.28/ 0.59 ±0.11)	0.67 (0.53/0.81 ±0.09)	0.49 (0.37/0.633 ±0.09)	0.46 (0.34/0.611 ±0.11)	0.60 (0.38/0.794 ±0.12)
TS (%)	1.52 (1.36/1.75 ±0.11)	1.53 (1.40 /1.67 ±0.10)	1.48 (3.14/1.83 ±0.17)	1.64 (1.52/1.84 ±0.10)	1.63 (1.48/1.79 ±0.10)	1.76 (1.62/1.86 ±0.07)
TOC/TN	2.27 (1.93 /3.76 ±0.56)	3.04 (2.17 /4.08±0.52)	3.29 (2.08 /7.60±1.65)	2.82 (1.88 /5.22±1.02)	2.87 (2.17 /5.57±1.00)	2.48 (1.81 /4.58±0.82)
TOC/TS	1.82 (1.05 /3.21±0.63)	2.04 (3.14 /1.12±0.77)	3.29 (1.84 /6.04±1.24)	1.97 (1.03 /3.62±0.89)	1.93 (1.04 /3.84±0.92)	1.98 (0.89 /3.57±0.86)
SO_4_^2-^(mM)	27 (24 /29±1.7)	24 (20 /30±3.1)	23 (20 /27±2.2)	27 (24 /29±1.7)	27 (24 /29±1.6)	26 (22 /29±2.8)
Chloride (mM)	0.15 (0.14/0.16 ±0.01)	0.15 (0.14/0.19 ±0.01)	0.14 (0.10/0.15 ±0.01)	0.15 (0.14/0.16 ±0.01)	0.16 (0.15/0.16 ±0.00)	0.16 (0.15/0.18 ±0.01)
H_2_S (mM)	0.15 (0 /0.25±0.1)	1.44 (0.05/3.09±1)	2.79 (0.03/4.51±1.6)	0.007 (0/0.04±0)	1.25 (0/3.48±1.1)	1.99 (0.07/4.97±1.7)
AVS (%)	0.07 (0.01 /0.15±0.05)	0.05 (0.1 /0.01±0.03)	0.04 (0 /0.13±0.03)	0.10 (0.05 /0.18±0.05)	0.10 (0.05 /0.22±0.06)	0.09 (0.03 /0.19±0.05)
CRS (%)	0.72 (0.52 /0.97±0.15)	0.58 (0.3 /0.83±0.16)	0.46 (0.32 /0.64±0.09)	0.74 (0.29 /1.4±0.31)	0.95 (0.6 /1.24±0.21)	0.87 (0.25 /1.69±0.44)

If we extend our analysis to the shallowest inland station, station S1 (< 7 m water depth; S1 Table), sulfide failed to reach the overlying waters throughout the measurement period from April to June. Moreover, station S1 was largely void of sulfide, or at least, when compared to stations S2 and S3 ~10 m water depth), suggesting that the shallowest shelf sediments are not contributors to ESP formation off the coast of Peru (Table 1; S4 Fig). From April to June, the water column at station S1 was oxygen-rich, which likely occurs as a result of the intensive mixing of the overlying water column (S3 Fig), providing greater ventilation to shelf sediments in this location.

Overall, if we combine our flux measurements in Paracas Bay with benthic sulfide fluxes reported by Sommer et al. 2016 from a transect further North (off roughly Callao), then we can constrain the potential ESP contributing shelf sediments to stations that are between 10–200 m depth. The area of the sediments that then contributes the most to ESP formation appears to be around 75 m depth, where the benthic sulfide flux can exceed 13 mmol m^-2^ d^-1^ [32].

### Sediment pore water and elemental composition

**Fig 5:**
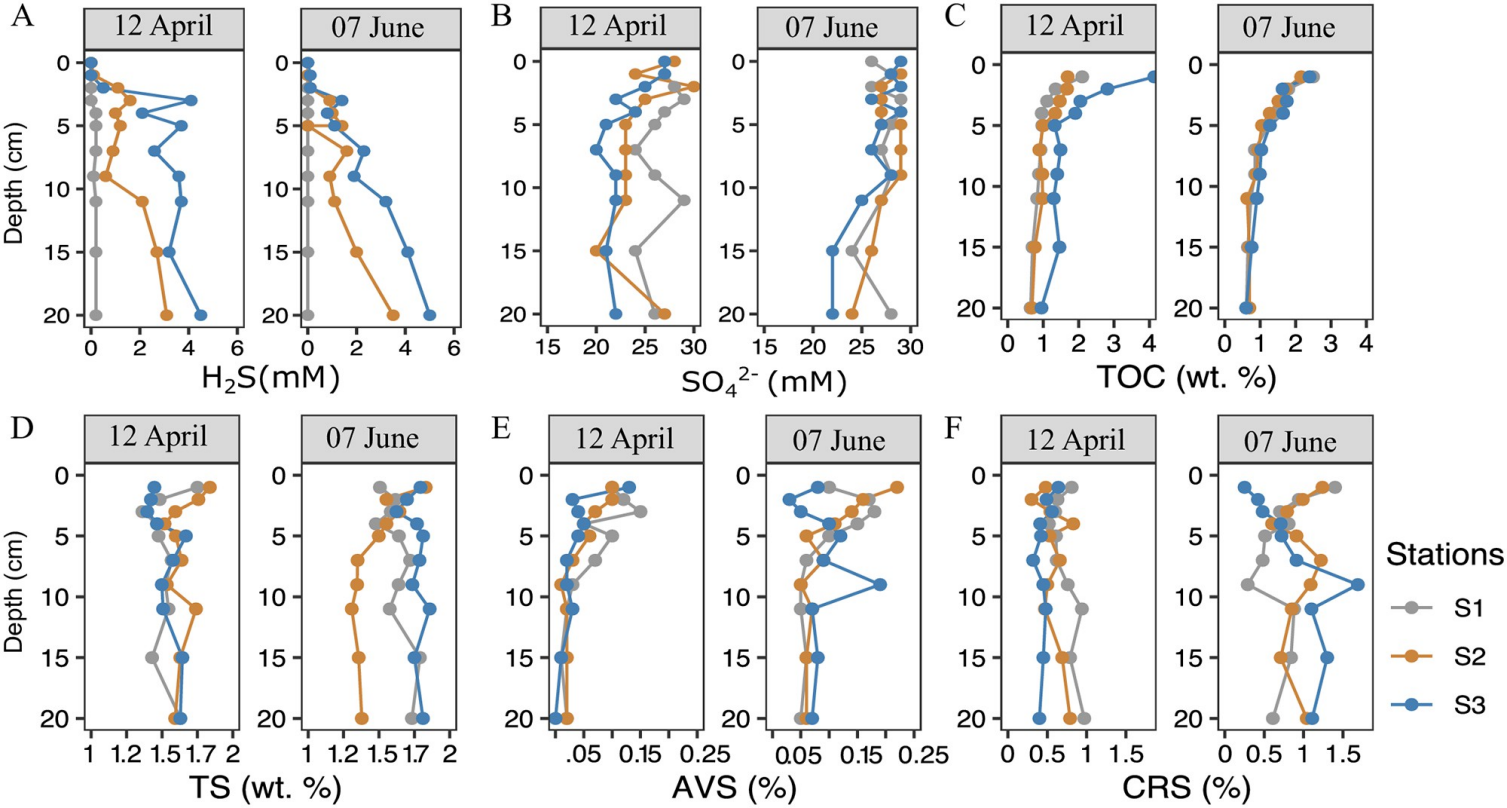
Pore water chemistry under anoxic (April, pre-El Nino) and oxic (July, post El Nino) conditions. Vertical distribution of pore water geochemical variables collected at stations S1, S2 and S3 include: (A) H_2_S, (B) SO_4_^2-^, (C) total organic carbon, (D) total sulfur, (E) acid volatile sulfides-AVS, (F) chromium reducible sulfur-CRS.

In April, the highly anoxic/sulfidic conditions at station S3, coincided with high concentrations of TOC in surface sediments (0–4 cm), reaching a maximum of 4% dry weight, which was nearly four-fold higher than maximum values reported at stations S2 (1.15% wt. %) and S1 (1.05 wt. %; Fig 5C; S3 Table). The positioning of station S3 slightly closer to the inflowing PCC surface current could possibly explain the higher rates of organic matter loading compared to the more coastal stations S2 and S1 (Fig 1A). Previous studies have shown that organic matter deposition rates can vary across the Peruvian upper shelf [38, 63]. In the shelf area from Callao to Pisco (12–15°S), the rates of organic matter loading are known to exceed 20 mmol m^-2^ d^-1^ –the cutoff for ESP formation [34]–whereas further North (> 11°S) the rates fall below this ESP-inducing threshold [38, 64]. Similarly, rates of sulfate-reduction tend to be greatest in the shelf area from Callao to Pisco [65]. In our study, while the deposition rates are not known, sufficient organic matter was available to drive a high benthic sulfide flux, and relatively sulfate-depleted conditions at station S3 (Fig 4B, 4D and 4F; S3 Table).

In June, following the oxygenation of the shelf, station S3 experienced a significant drop in the TOC pool from 0–4 cm, decreasing by as much as 1.7%, from values observed in April (Fig 5C; S3 Table). No significant changes were observed at station S2. Moreover, the decline in TOC concentrations at stations S2 and S3 in June caused them to resemble the vertical TOC profiles observed at station S1 (oxygen-rich water column; Fig 5C). The arrival of the El Niño oxygenation event had substantially lowered TOC levels, likely by means of enhancing organic matter respiration. While the drop in TOC concentrations was most notable in the superficial sediment layer due to its close contact with oxygen-rich water, a moderate decrease of nearly 0.9% was also evident in deeper layers of the sediment from 4–20 cm. Changes in deeper inventories of TOC are difficult to reconcile without invoking mechanisms such as macrofauna-induced bioirrigation. Previous work has shown that increases in macrofauna re-colonization/bioirrigation occur after the arrival of El Niño driven oxygenation events [39]. The presence of macrofauna and their burrow networks are known to accelerate organic matter remineralization rates in deep sediments [66].

The oxygenation period also had an impact on the accumulation of iron-bound sulfur species AVS (Fe monosulfide) and CRS (primarily FeS_2_). Across the entire sediment interval, AVS content ranged from 0 to 0.22%, with AVS levels being greatest in the upper sediment layer and decreasing with depth (Fig 5E). Whereas, CRS content, while generally an order of magnitude higher than AVS concentrations, showed no clear vertical distribution pattern in the top 20 cm of the sediment (Fig 5F, S3 Table). Overall, the reported range of AVS and CRS values were consistent with Peruvian shelf sediments observed by Fossing [20], and in the same order of magnitude as values reported in Brocławik et al. [67]. Most striking, however, was that sediment cores collected at stations S2 and S3 in June had greater CRS and AVS content by as much as 64% and 100% compared to cores analyzed in April (Table 1). Likewise, sediment TS content, which mostly reflects iron-bound sulfur, increased by as much as 7–19% at stations S2 and S3, between April and June (Table 1, Fig 5D). From a depth perspective, the transition from April to June was also associated with an increase in the variability of the CRS and AVS vertical structure. This was especially apparent in deeper layers of the sediment (> 5 cm) where values would increase by as much as 9.5 and 3.5-fold when compared between cores collected in April and June, for AVS and CRS, respectively (Fig 5E and 5F). As mentioned above, we attribute the large fluctuations in CRS/AVS values at deeper depths to the re-colonization of macrofauna that follow re-oxygenation events (e.g., El Niño) [39], along with the subsequent reworking of the sediments by these communities.

**Fig 6:**
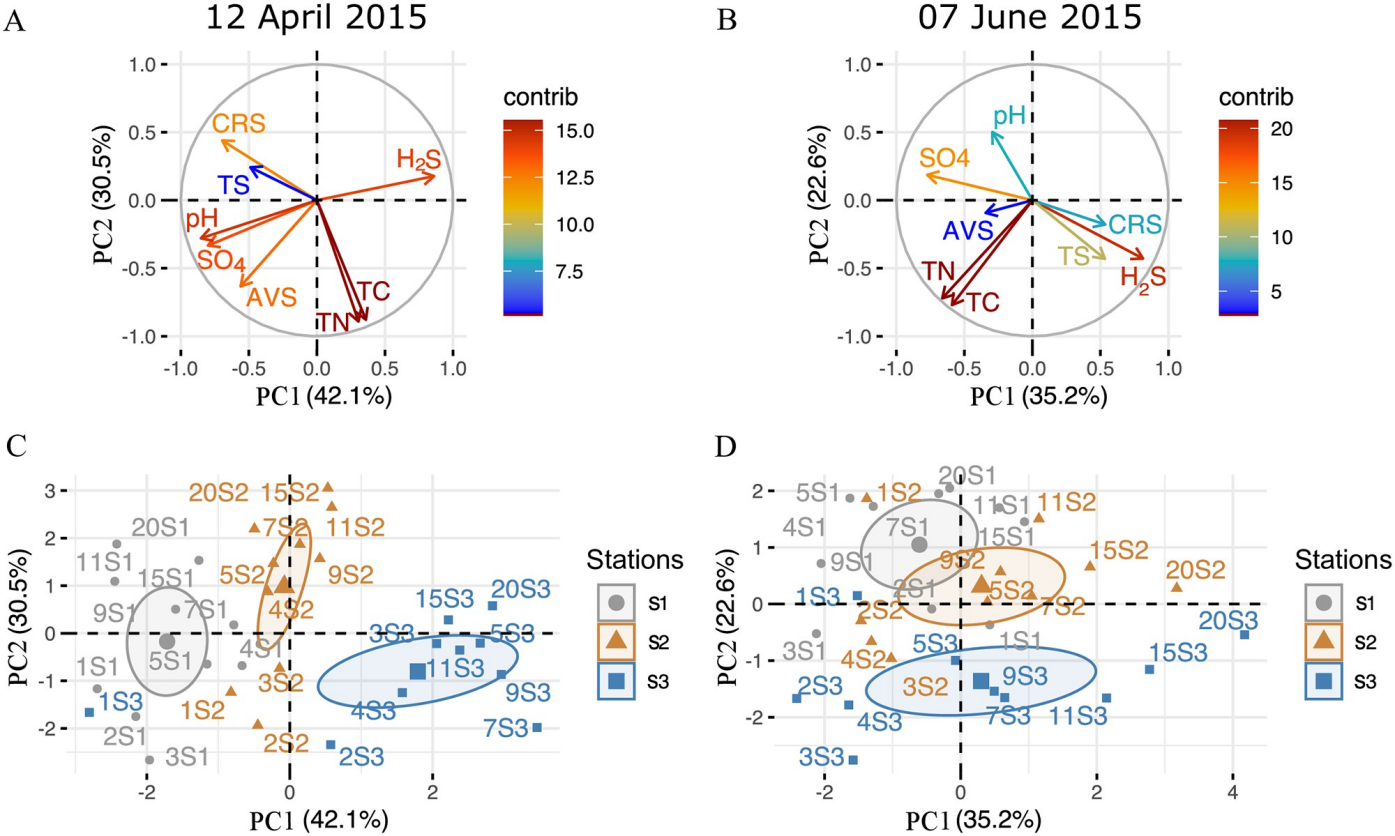
Geochemical shift in pore water chemistry during the transition from anoxic to oxic conditions following the arrival of the El Nino event. The principal component analysis (PCA) was performed on geochemical parameters measured from sediment cores representing two contrasting conditions: anoxic (April, pre-El Nino) and oxic (July, post El Nino). In (A, C) two components exhibit 72.6% of the total variance PC1 (42.1%) and PC2 (30.5%). In (B, D) two components exhibit 58.7% of the total variance PC1 (35.2%) and PC2 (22.6%). In (C, D) the ellipses represent 95% confidence intervals for each of the stations S1, S2 and S3. Abbreviations stand for: TC, total carbon; TN, total nitrogen; TS, total sulfur; AVS, Acid Volatile Sulfides; CRS, chromium reducible sulfur.

Overall, a distinguishable shift occurred for many of the measured parameters in Paracas Bay during the transition from anoxic (April) to oxic (June). Most notably, following the oxygenation, parameters such as TOC and sulfate at stations S2 and S3, converged to more closely resemble the biogeochemistry at station S1; while other parameters including AVS, CRS, sulfide and TS, showed the reverse trend (i.e., divergence) (Fig 5). This finding is also reflected in our PCA analysis (Fig 6A–6D), which finds a collation of station points along the x-axis before the El Niño event, followed by a clear shift and re-alignment of points along the y-axis post water column oxygenation (Fig 6C and 6D). Despite the lag in response that can exist between the oxygen fluctuations and the variables measured in the sediment, we observed changes in the first 10 cm where H_2_S and organic composition (i.e. TC, TN) contribute significantly to the explained variance (Fig 6A and 6B). While sediment core analyses were not performed throughout the entire anoxic period; we believe that the conditions observed in April provide some insight into conditions that were likely encountered throughout the anoxic period (i.e., in March) when ESP development was prominent.

### Prevailing hydrodynamic conditions

**Fig 7:**
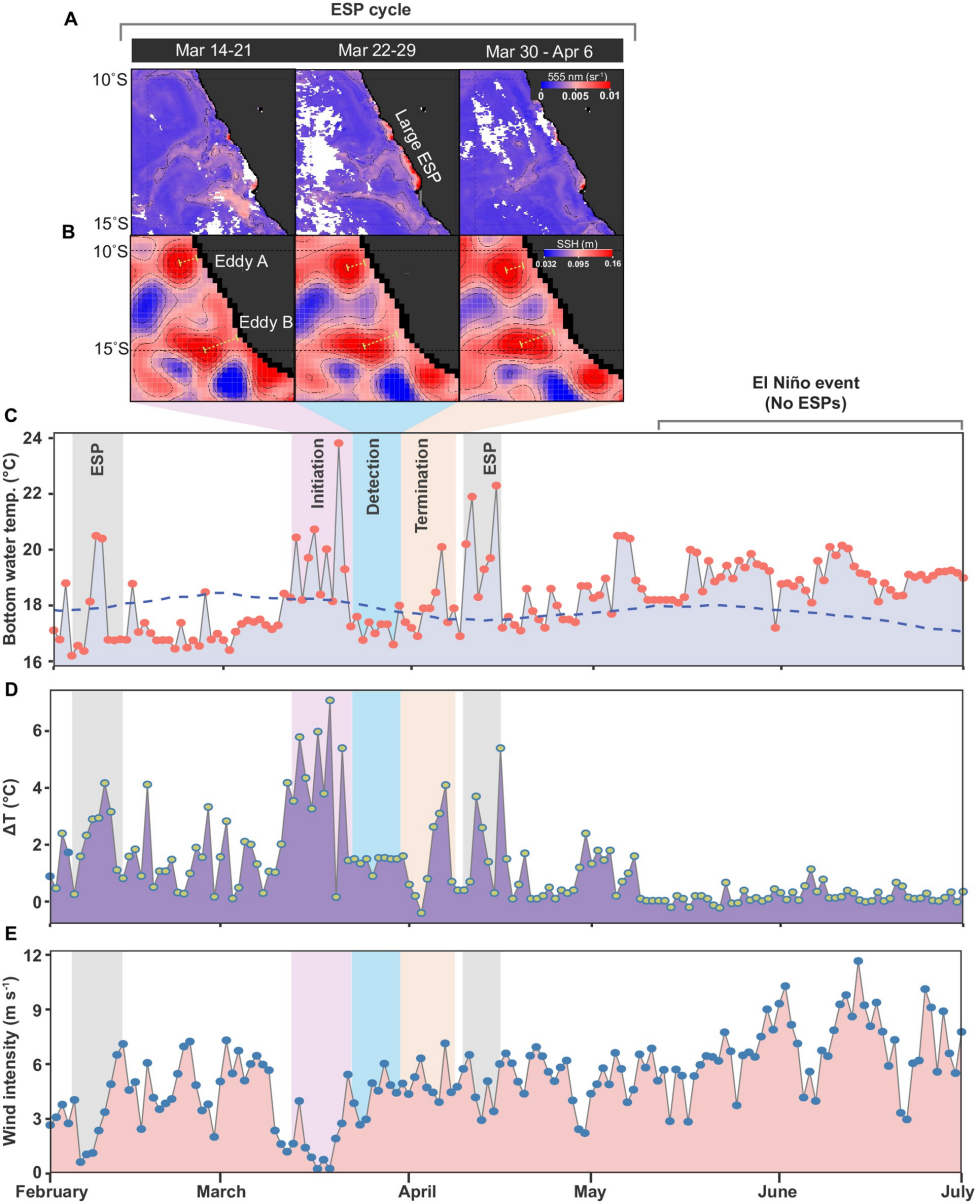
Tracking the development and termination of an elemental sulfur plume and its relation to other oceanographic conditions. The images/data recordings cover the austral summer-autumn period in 2015, off the coast of Peru. (A) Weekly composite MODIS images showing the development of the March-ESP event in near-surface waters between Callao and Pisco (weeks 1–3). The images show the water reflectance wavelength at 555 nm, which is the optimal band for elemental sulfur plume detection. (B) Weekly composite images of sea surface height altimetry (obtained from AVISO), indicating the presence of two anticyclonic mesoscale eddies (designated eddy A and eddy B) forming adjacent to the ESP event. Eddies A and B formed, and subsequently propagated off the coast from weeks 1 to 3. (C) Daily records of bottom water temperature near the San Martin Harbor (indicated by the blue star in Fig 1A), with the dashed blue line representing climatology values from 2006 to 2015. The colored rectangles with “initiation, detection and termination” are referring to the progression of the sulfur plume observed in panel A, and is also described in-text. (D) Difference between surface and bottom water temperature (ΔT °C), which is used here as a proxy of water column stratification according to Aguirre-Velarde et al., 2019. Note that panels C and D were modified from Merma [46]. (E) Daily coastal wind intensity (ms^-1^) acquired by Advanced Scatterometer (ASCAT) (see red star in Fig 1A).

We paired our biogeochemical analysis with a hydrodynamic investigation into the factors that govern ESP formation and termination. Namely, we examined the March ESP (Fig 2A), which had propagated over a 3-week timespan from March 14 to April 6, according to the remote sensing of sulfur plumes (Fig 7A). To this end, we examined how changes in coastal wind, water temperature, and sea surface height may have contributed to the March ESP event between Callao and Pisco.

The tracked ESP event in March, which was readily detectable in surface waters between Callao and Pisco from March 22 to 29 (in week 2; Fig 7A), preceded a bottom water temperature anomaly (in week 1; Fig 7C and 7D). This temperature anomaly, which shifted bottom water temperatures by ~7°C above background levels (maximum of ~20°C), persisted over 10 days. Moreover, the temperature anomaly coincided with a 6-fold weakening of coastal wind intensities (in both the alongshore and cross-shore directions; S2 Fig), with wind speeds decreasing from 6 m s^-1^ (ambient) to less than 1 m s^-1^ (Fig 7E). Eventually, both wind speeds and bottom water temperatures returned to background levels after March 24 (Fig 7C and 7E). The onset of the anomalous temperature shift on March 14 to 21 (week 1) did not immediately manifest in the formation of a surface-detected sulfur plume at the coast; however, a large ESP would occur in the following week from March 22 to 29 (week 2), with the plume later dissipating from March 30 to April 6 (week 3).

Similar anomalies in sea surface temperature and a weakening of mean wind intensities (Fig 2C and 2D) were observed in both early February and mid-April, albeit, the duration, size, and intensity of the anomalies were comparatively small compared to the March ESP event. Specifically, the resulting sulfur plume detected on April 18^th^ (Fig 1B) was preceded by a 6-day period that was associated with an increase in sediment-overlying bottom water temperature (~22°C), and a temperature change from background levels attaining up to ~5°C (Fig 7C and 7D). Moreover, the 6-day temperature anomaly was accompanied by a brief drop in wind intensities. Overall, our cross-correlation function (CCF) analysis between the time series of wind intensity and sediment-overlying bottom water temperature finds a weak positive correlation (0.34, p-value < 0.05). Whereas the wind intensity and water column stratification proxy (ΔT °C) (Fig 7D) showed a higher negative correlation (-0.48, p < 0.05) with a 1-day lag, over the study period (S5 Table). In other words, decreasing wind intensities were significantly associated with increasing water column stratification (S5 Fig).

Sea surface height altimetry would provide additional information, concerning the presence or absence of mesoscale eddies, which have been previously hypothesized to play a role in ESP development [2, 13]. It should be noted that cyclonic and anticyclonic eddies generate negative and positive sea surface height anomalies, respectively. Interestingly, during the March ESP event, two anticyclonic mesoscale eddies (designated Eddy A and Eddy B) had formed adjacent to the shallow upper shelf waters between 10–15°S (Fig 7B). Previous studies have shown that eddies commonly develop between 10–15°S due to the topographical coastal bends that create instabilities in the PCUC [36, 40, 42]. In our study, Eddy A had formed off the coast of Callao, while Eddy B was situated 70 kilometers South, and near Paracas Bay peninsula (Fig 7B). Eddies A and B were approximately 40 and 48 km^2^ in diameter, respectively. Both eddies had originated near the coast, with the eddy center located at 16 and 28 km from the coast on March 14–21 (week 1), respectively. Moreover, the occurrence of the eddies in this region was also evidenced by the presence of offshore chlorophyll and sulfur plume filaments, which extended up to 250 km into the open ocean (Fig 2A and 2B). Notably, the period when the eddies were closest to the coast also coincided with low wind intensities (Fig 7E), and warm sea surface temperature anomalies in Paracas Bay (Fig 7C and 7D). Following the increase in wind speeds (in both the cross-shore and alongshore directions; S2 Fig), the Eddies A and B would begin their propagation westward on March 22–29 (week 2), reaching 20 and 36 km from the coast, respectively. The separation of eddies A and B from the shelf continued into week 3 (March 30 to April 6), reaching 24 and 40 km from the coast, respectively (Fig 7B). Overall, the eddies propagation westward appeared to also coincide with the dissipation of the March ESP (Fig 7A), further implicating their role in ESP development and termination (discussed below).

## Discussion

### The biogeochemical conditions that gave rise to the March 2015 sulfidic event

The ESP tracked in March was one of the largest events reported in the Peruvian upwelling zone, comprising an area of 17,000 km^2^ and lasting 2 to 3 weeks. The March ESP event, which had formed in shallow coastal waters (< 200 m depth), was later dispersed into the open ocean. Below we discuss the myriad of biogeochemical parameters, obtained from our water column and sediment analyses that likely contributed to its development. It should be noted that many of these factors are not necessarily unique to our tracked ESP, but share some resemblances with previously documented events in the ETSP region [8, 11, 13, 29], and with others reported off the coast of Namibia and India [5, 9, 23, 31, 33, 35,].

Organic matter is central to the sulfur cycle. Like other events, our ESP occurred in the austral summer/autumn when surface primary productivity is greatest. ETSP studies have shown that the transition from winter to summer marks a fivefold increase in the organic matter export rates to the sediment, from 3 ± 2 to 17 ± 10 mmol m^-2^ d^-1^, respectively [34, 38, 68]. ESP events tend to be most favorable when organic matter export rates exceed 20 mmol m^-2^ d^-1^ [34]. Furthermore, the organic-rich conditions in the sediment are likely to enhance anoxia, contributing further to the preservation of organic material deposited in the sediment [38]. Adding to this body of work, we show that under anoxic/sulfidic conditions (in April) intensive organic matter accumulation occurred at our study site, especially when juxtaposed against the same sediments but sampled after an oxygenation event had taken place (in June). This large preservation of organic material under anoxic conditions was likely to have provided substantial fuel for heterotrophic sulfate-reducing bacteria, explaining the correspondingly high benthic sulfide flux (4.3 mmol m^-2^ d^-1^) and relatively high sulfide concentrations in surface sediments. Previous studies have reported ETSP sulfate-reduction rates reaching up to hundreds of nmol cm^-3^ d^-1^, which are in large part driven by the massive inventory of organic matter [69].

The benthic sulfide during the anoxic period was subsequently oxidized by available bottom water nitrate. Due to the oxygen-depleted nature of the ETSP upwelling zone (e.g. Fig 3A), nitrate usually remains as the main oxidant of benthic-generated sulfide. Analogous to other documented ETSP-ESPs, our study period was characterized by a general depletion of both nitrate (< 1 μM) and oxygen (< 20 μM) concentrations in bottom waters. The general absence of nitrate in bottom waters is maintained as a result of the activities of sulfide-oxidizing nitrate-reducing bacteria, such as *Arcobacter peruensis* and ^*U*^*Thioglobus perditus* (SUP05; “U” indicates an uncultivated taxon) [11, 13, 28, 70]. In addition, sediment communities of filamentous sulfide-oxidizing bacteria *Thioploca* (*Ca*. Venteria ishoeyi) may also contribute to the uptake of water column nitrate, which they can store inside intracellular vacuoles [50, 51, 53]. These organisms have the potential to generate elemental sulfur as an intermediate of sulfide oxidation coupled to nitrate reduction, which is the intermediate sulfur species that are detected by satellite remote sensing. Microbes, such as *Thioploca* and *Thioglobus*, can even store elemental sulfur inside intracellular vacuoles for later oxidation to sulfate when sulfide concentrations become limiting (i.e. after the ESP subsides). Our estimates suggest that to fully oxidize the benthic sulfide flux (4.3 mmol m^-2^ d^-1^) to sulfate, then the counteracting nitrate flux would need to exceed 6.9 mmol m^-2^ d^-1^ (according to the stoichiometry of sulfide-oxidizing nitrate reducing below):

$$5{\text{H}}_{2}\text{S}+8{\text{NO}}_{3}{}^{-}\to 5{\text{SO}}_{4}{}^{2-}+4{\text{N}}_{2}+4{\text{H}}_{2}\text{O}+2{\text{H}}^{+}$$

If the nitrate replenishment rate falls below 6.9 mmol m^-2^ d^-1^ then sulfide is expected to accumulate in the water column. Given the size of the March ESP, the plume was likely responsible for oxidizing a significant amount of nitrate to N_2_ gas–contributing to substantial nitrogen loss from a singular event. It is worth noting that the H_2_S fluxes detected in our study are lower than those reported by Brüchert et al. [71] in the central Namibian coastal upwelling zone (approximately 2–11 mmol m^-2^ d^-1^) and by Dale et al. [6] on the Peruvian margin (approximately 12 mmol m^-2^ d^-1^), where NO_3_^-^ and NO_2_^-^ were absent.

Perhaps one of the most striking observations of this study is the accumulation of AVS (Fe monosulfide), and CRS (i.e., FeS_2_) in June under oxic conditions, then compared to April when anoxia prevailed over the shelf (Figs 5D and 7C). We believe that the re-oxidation of the sediment, following the arrival of the El Niño event, oxidized available iron that, in turn, precipitated with sulfide to account for the rise in AVS and CRS inventories between April and June. In addition, TS, which mainly reflects iron-bound sulfur, showed the same pattern. The presence of AVS, and CRS, which can serve as a proxy for the availability of reactive Fe (II) [72], suggest that reactive iron could play an important role in H_2_S sequestration in ETSP sediments. Indeed, the iron flux from shelf sediments is widely recognized as an important source of Fe in coastal waters [72, 73]. Overall, the presence of reactive iron may to some extent buffer the benthic sulfide flux in ETSP waters, with similar findings also in other sulfide-rich sediments off the coast of Namibia and India [23, 53, 71].

### Local wind forcing and how it impacts ESP development

While biogeochemical conditions favored ESP formation in austral summer, hydrodynamic conditions also played a key role in ESP propagation. Previous studies have emphasized the role of water column stagnation/stratification, upwelling intensity, El Niño/La Nina events, and monsoon rains as factors contributing to ESP development off the coast of Peru, Chile, Namibia, and India [5, 9, 8, 11, 13, 23, 29, 31, 35]. Some of these hydrodynamic features inducing ESPs are shared between upwelling regions, while others are unique to certain systems. Most notably, monsoonal rains or major rain events are not considered key drivers of ESP development in the ETSP region, given its arid nature. Whereas other factors inducing ESPs, such as changes in upwelling intensity and stratification and their linkage to shifts in wind patterns were subjects of greater investigation here. The low intensity of winds and the corresponding increase in temperature (Fig 7C–7E) can lead to a reduction in upwelling intensity, resulting in greater stratification of the water column. We can speculate about how stratification hinders the mixing of bottom waters, promoting the remineralization of organic matter through sulfate reduction processes, which supports the release of H_2_S from sediments. Stratification can also stimulate phytoplankton growth, providing labile material to bottom waters and triggering oxygen consumption. These processes could occur simultaneously and favor the formation of ESP.

Our discussion below, in large part, focuses on the hydrodynamic features and their impact on the intensity of the PCUC. The PCUC is the main current responsible for nutrient upwelling and elevated primary productivity in surface waters. In addition, the PCUC is in close contact with the upper and lower shelves between 100–150 m depth [40]–where the benthic sulfide flux is greatest–and therefore serves a key role in ESP formation and termination [32, 54].

In our tracking of the March ESP, we find that attenuation in local wind intensity was ultimately key to its formation during the austral summer-autumn period. The drop in wind intensities was further accompanied by an increase in sea surface temperatures; suggesting that after the winds had subsided the PCUC upwelling of cold waters was temporarily attenuated during this same period. Off the coast of Namibia, a long-term ESP survey conducted over seasonal and interannual timeframe reported that wind intensity shares a negative relationship with sulfur plume intensity [29]. In other words, the authors demonstrated that as wind intensities decreased, the overall size of the sulfur plume increased. Furthermore, Ohde et al., [29], found that larger ESPs occurred most often when sea surface temperatures were relatively warm, alluding to the possibility that the upwelling of cool deep waters was weakened over the same low wind intensity period. The long-term ESPs survey in Namibia, therefore, shares interesting parallels to our case study event tracked off the coast of central Peru.

While the interruption in wind intensity was a likely contributor to ESP formation, some questions persist as to why the ESP event was detected 1-week after wind intensities had increased and sea surface temperatures cooled to pre-event levels. This unusual lag period could be partly attributed to the time it takes to accumulate elemental sulfur via biological sulfide oxidation in bottom waters. Moreover, even if sulfur has accumulated in bottom waters, it is unlikely to be detected by remote sensing until the water mass is upwelled to surface waters. In our remotely detected ESP event, we believe that the sulfur-rich water mass (found in close contact with the benthos) was upwelled to the surface waters (via the PCUC) shortly after the wind intensities had resumed to background levels. The resumption of the PCUC-driven upwelling was also evidenced by the introduction of cooler waters to the surface, coinciding with our remotely detected ESP event. Interestingly, in the ESPs detected off the coast of Namibia, [31] observed a similar phenomenon: singular ESP events became detectable by remote sensing after upwelling conditions had intensified. Others have suggested that some events may even go undetected if the elemental sulfur is not upwelled, partly biasing surveys investigating ESP frequency [5].

Other factors may further complicate ESP development in the ETSP region. For example, the development of mesoscale eddies A and B, and their close association with the upper shelf when the March ESP occurred, raised additional questions regarding their role in ESP development. In ETSP surveys, mesoscale eddies are known to impact the PCUC by causing the fast-moving current to meander around coastal forming eddies [40]. In some cases, interruptions in the PCUC can last for days to weeks [40]. Later work by Thomsen et al. [36] indicates that as the PCUC is temporally diverted around the eddy, lower current velocities ensue over the adjacent upper shelf, forming a so-called ‘shadow zone’. The subverted PCUC, resulting in a near stagnant water column, and the lack of nitrate replenishment to the shelf, was ostensibly linked to the formation of an ESP, which had developed in the ‘shadow zone’ adjacent to the eddy over the same period [13]. We believe that a similar scenario may have led to the March 2015 ESP, following the development of eddies A and B over the shelf. Adding to this scenario, the week-long attenuation of the winds, in mid-March, was likely to have further constrained the eddies’ propagation westward, thereby prolonging its association with the shelf. It is therefore tempting to speculate that mesoscale hydrodynamics add another level of complexity to ESP formation in the ETSP region. Given the frequency of eddies in ETSP waters, we hope that eddy-resolving hydrodynamic and biogeochemical coupled models will provide greater insight into the exact mechanism.

What is clear from the remote sensing of Eddies A and B, is that as the anticyclonic eddies turned, elemental sulfur was advected offshore (> 250 km) along its northern rim as evidenced by the multiple offshore protruding filaments (in early to mid-March). Previous in situ measurements of eddy-advected filaments have found that these features carry coastal temperature-salinity characteristics, and are rich in both elemental sulfur and sulfide-oxidizing bacteria [13]. The sulfide-oxidizing bacteria were further shown to have additional ecophysiological features (i.e., sulfur storage) that enable their survival in advected offshore filaments. The eventual dissipation of the offshore advected filament, and the main coastal ESP itself, occurred after both eddies A and B, commenced their westward propagation (from March 22 onward). The westward propagation would be driven by the increase in the cross-shore winds, which had intensified over this same period (S2 Fig). Previous work by Thomsen et al. [36] revealed that as an eddy propagates away from the coast, the previously subverted PCUC current then resumes its original coastal shelf track. The rapid increase in bottom water current velocities and the replenishment of nitrate over the upper shelf mitigate any further benthic fluxes of sulfide and cause the remainder of the sulfur plume to be dispersed. Altogether, the entire life cycle of the ESP (ca. 2–3 weeks), from formation to termination, would last the duration of the eddy’s life cycle over the shelf, revealing a close linkage.

Overall, our findings suggest that the attenuation of local wind is a key driver of ESP development. Namely, attenuations in the local wind constrain upwelling intensity driven by the PCUC. In addition, interruptions in the local wind may place auxiliary constraints on wind-driven mesoscale eddy propagation from the coast. Both these hydrodynamic mechanisms potentially moderate the PCUC intensity either directly or indirectly, with downstream consequences for ESP development. Furthermore, these hydrodynamic conditions underpin overall shelf biogeochemistry and surface primary productivity driving the highly active sulfur cycle in the ETSP region. While we offer some insight into ESP development from our case study, we hope that future studies widen their investigation to include a more comprehensive timeframe. Lastly, we suggest that local wind patterns could serve as a diagnostic tool for predicting ESP development in oxygen-depleted marine environments.

## Conclusions

Elemental sulfur plumes can be observed off the central coastal zone of Peru during the austral summer. This occurs when the water above the upper shelf has low oxygen and nitrate levels, and the shelf sediments are rich in organic matter but low in iron-bound sulfur. Local wind interruptions constrain upwelling intensity, resulting in increased stratification over the upper shelf that serves as a trigger for elemental sulfur plumes. However, the arrival of an El Niño event can disrupt this process by transporting oxygen. The presence of reactive iron may help sequester H_2_S, thus limiting the development of elemental sulfur plumes. This study highlights the sensitivity of the coastal zone to changes in dissolved oxygen and the potential consequences of sulfide fluxes in the context of hypoxia expansion in coastal ecosystems.

## Supporting information

S1 Fig Determining the size of the elemental sulfur plume observed off the coast of Peru in March 2015. (A) MODIS images of the elemental sulfur plume as detected by the water reflectance wavelength at 555 nm (optimal band for ESP detection). (B) Pixel histograms collected for the water reflectance wavelength at 555 nm. The ESP event(s) detected in February and March were denoted by the presence of high intensity sulfur-containing pixels (indicated by the grey arrows), resulting in a bimodal histogram profile. The March bimodal histogram is especially distinct from the May monomodal histogram profile, which represents a non-ESP scenario (i.e., post El Nino). As discussed in the methods, we performed a background excess calculation, in that, the binned pixels (at 555 nm) associated with a non-ESP month (May) were subtracted from the binned sulfur-containing pixels of the March 2015 period (ESP scenario). Given the known area of a pixel, we deduced that the March ESP was an estimated 16,963 km^2^.
(PDF)

S2 Fig Daily time series showing changes in alongshore (red) and cross-shore (blue) wind velocities in the ETSP region. The wind velocity data was acquired by Advanced Scatterometer (ASCAT) aboard Metop-A and Metop-B satellites, during the austral summer-autumn period in 2015, off the coast of Peru (see also Fig 1A).
(TIF)

S3 Fig Dissolved bottom water oxygen concentrations off the coast of Peru. Synoptic time series images were taken during the austral summer-autumn period in 2015. In both panels (A, B), the blue line denotes the smoothed daily mean, while the grey shading illustrates the confidence interval. The red line indicates the dissolved oxygen threshold for suboxic (< 20 μM) and dysoxic (20–90 μM) conditions, as previously defined according to Wright [42].
(TIF)

S4 Fig Vertical and temporal profiles of pore water sulfate and sulfide concentrations from sediment cores collected in Paracas Bay. Pore water concentrations of (A) sulfide, (B) sulfate, and (C) sulfate/chloride ratio are shown. Note that the arrival of the El Nino event (i.e., re-oxygenation of the water column) occurred from May 17 onward. Prior to the El Nino event, the water column was largely void of both oxygen and nitrate, see also Fig 3A–3C.
(TIF)

S5 Fig Water-column temperature (°C) over Paracas Bay. Temperature (°C) measured in situ, linearly interpolated to 1 m depth intervals at the three stations (S1, S2, & S3). The black box shows the arrival of the 2015 El Niño event.
(TIF)

S1 Table Locations and sampling resolution at Paracas Bay. Abbreviations stand for: TIC, total inorganic carbon; TC, total carbon; TN, total nitrogen; TS, total sulfur; AVS, Acid Volatile Sulfides; CRS, chromium reducible sulfur.
(XLSX)

S2 Table Pore water sulfate and sulfide concentrations at the three Paracas Bay sites (S1, S2 & S3). (XLSX)

S3 Table Biogeochemical variables of solid phase in sedimentary components. (XLSX)

S4 Table Input data from Paracas Bay for diffusive flux calculations. (XLSX)

S5 Table Estimation and significance testing of cross-correlation between environmental parameters with positive time-lags. (XLSX)

## References

[1] Wasmund K, Mußmann M, Loy A. The life sulfuric: microbial ecology of sulfur cycling in marine sediments. Environmental microbiology reports. 2017;9: 323–344. doi: 10.1111/1758-2229.12538 28419734 PMC5573963

[2] Callbeck CM, Canfield DE, Kuypers MM, Yilmaz P, Lavik G, Thamdrup B, et al. Sulfur cycling in oceanic oxygen minimum zones. Limnology and Oceanography. 2021.

[3] Canfield DE, Stewart FJ, Thamdrup B, De Brabandere L, Dalsgaard T, Delong EF, et al. A cryptic sulfur cycle in oxygen-minimum-zone waters off the Chilean coast. Science. 2010;330: 1375–1378. doi: 10.1126/science.1196889 21071631

[4] van Vliet DM, von Meijenfeldt FB, Dutilh BE, Villanueva L, Sinninghe Damsté JS, Stams AJ, et al. The bacterial sulfur cycle in expanding dysoxic and euxinic marine waters. Environmental Microbiology. 2021;23: 2834–2857. doi: 10.1111/1462-2920.15265 33000514 PMC8359478

[5] Lavik G, Stührmann T, Brüchert V, Van Der Plas A, Mohrholz V, Lam P, et al. Detoxification of sulphidic African shelf waters by blooming chemolithotrophs. Nature. 2009;457: 581–584. doi: 10.1038/nature07588 19078958

[6] Dale AW, Sommer S, Lomnitz U, Bourbonnais A, Wallmann K. Biological nitrate transport in sediments on the Peruvian margin mitigates benthic sulfide emissions and drives pelagic N loss during stagnation events. Deep-Sea Research Part I: Oceanographic Research Papers. 2016;112: 123–136. doi: 10.1016/j.dsr.2016.02.013

[7] Noffke A, Hensen C, Sommer S, Scholz F, Bohlen L, Mosch T, et al. Benthic iron and phosphorus fluxes across the Peruvian oxygen minimum zone. Limnology and Oceanography. 2012;57: 851–867. doi: 10.4319/lo.2012.57.3.0851

[8] Schunck H, Lavik G, Desai DK, Großkopf T, Kalvelage T, Löscher CR, et al. Giant Hydrogen Sulfide Plume in the Oxygen Minimum Zone off Peru Supports Chemolithoautotrophy. PloS ONE. 2013;8. doi: 10.1371/journal.pone.0068661 23990875 PMC3749208

[9] Naqvi S, Jayakumar D, Narvekar P, Naik H, Sarma V, D’souza W, et al. Increased marine production of N2O due to intensifying anoxia on the Indian continental shelf. Nature. 2000;408: 346–349. doi: 10.1038/35042551 11099038

[10] Shenoy DM, Sujith K, Gauns MU, Patil S, Sarkar A, Naik H, et al. Production of dimethylsulphide during the seasonal anoxia off Goa. Biogeochemistry. 2012;110: 47–55.

[11] Galán A, Faúndez J, Thamdrup B, Santibáñez JF, Farías L. Temporal dynamics of nitrogen loss in the coastal upwelling ecosystem off central Chile: Evidence of autotrophic denitrification through sulfide oxidation. Limnology and Oceanography. 2014;59: 1865–1878.

[12] Lomnitz U, Sommer S, Dale AW, Löscher CR, Noffke A, Wallmann K, et al. Benthic phosphorus cycling in the Peruvian oxygen minimum zone. Biogeosciences. 2016;13: 1367–1386.

[13] Callbeck CM, Lavik G, Ferdelman TG, Fuchs B, Gruber-Vodicka HR, Hach PF, et al. Oxygen minimum zone cryptic sulfur cycling sustained by offshore transport of key sulfur oxidizing bacteria. Nature communications. 2018;9: 1–11. 10.1038/s41467-018-04041-x PMC5928099 29712903

[14] Shirodkar G, Naqvi SWA, Naik H, Pratihary AK, Kurian S, Shenoy DM. Methane dynamics in the shelf waters of the West coast of India during seasonal anoxia. Marine Chemistry. 2018;203: 55–63.

[15] Hamukuaya H, O’Toole M, Woodhead P. Observations of severe hypoxia and offshore displacement of Cape hake over the Namibian shelf in 1994. South African Journal of Marine Science. 1998;19: 57–59.

[16] Cockcroft AC. Jasus lalandii’walkouts’ or mass strandings in South Africa during the 1990s: an overview. Marine and Freshwater Research. 2001;52: 1085–1093.

[17] Monteiro PMS, van der Plas AK, Mélice JL, Florenchie P. Interannual hypoxia variability in a coastal upwelling system: Ocean-shelf exchange, climate and ecosystem-state implications. Deep-Sea Research Part I: Oceanographic Research Papers. 2008;55: 435–450. doi: 10.1016/j.dsr.2007.12.010

[18] Copenhagen W. The periodic mortality of fish in the Walvis region. South African Medical Journal. 1954;28: 381. 13168630

[19] Levin L, Ekau W, Gooday A, Jorissen F, Middelburg J, Naqvi S, et al. Effects of natural and human-induced hypoxia on coastal benthos. Biogeosciences. 2009;6: 2063–2098.

[20] Fossing H. Sulfate reduction in shelf sediments in the upwelling region off Central Peru. Continental Shelf Research. 1990 pp. 355–367. Report No.: 4.

[21] Ferdelman TG, Lee C, Pantoja S, Harder J, Bebout BM, Fossing H. Sulfate reduction and methanogenesis in a Thioploca-dominated sediment off the coast of Chile. Geochimica et Cosmochimica Acta. 1997;61: 3065–3079.

[22] Ferdelman TG, Fossing H, Neumann K, Schulz HD. Sulfate reduction in surface sediments of the southeast Atlantic continental margin between 1538S and 2757S (Angola and Namibia). Limnol Oceanogr. 1999 pp. 650–661. Report No.: 3.

[23] Brüchert V, Jørgensen BB, Neumann K, Riechmann D, Schlösser M, Schulz H. Regulation of bacterial sulfate reduction and hydrogen sulfide fluxes in the central Namibian coastal upwelling zone. Geochimica et Cosmochimica Acta. 2003;67: 4505–4518. doi: 10.1016/S0016-7037(03)00275-8

[24] Fernandes S, Mazumdar A, Peketi A, Anand SS, Rengarajan R, Jose A, et al. Sulfidization processes in seasonally hypoxic shelf sediments: A study off the West coast of India. Marine and Petroleum Geology. 2020;117: 104353.

[25] Mazumdar A, Peketi A, Joao H, Dewangan P, Borole D, Kocherla M. Sulfidization in a shallow coastal depositional setting: Diagenetic and palaeoclimatic implications. Chemical Geology. 2012;322: 68–78.

[26] Schlosser C, Streu P, Frank M, Lavik G, Croot PL, Dengler M, et al. H2S events in the Peruvian oxygen minimum zone facilitate enhanced dissolved Fe concentrations. Scientific Reports. 2018;8: 1–8. doi: 10.1038/s41598-018-30580-w 30140004 PMC6107642

[27] Scholz F, McManus J, Mix AC, Hensen C, Schneider RR. The impact of ocean deoxygenation on iron release from continental margin sediments. Nature Geoscience. 2014;7: 433–437.

[28] Callbeck CM, Pelzer C, Lavik G, Ferdelman TG, Graf JS, Vekeman B, et al. Arcobacter peruensis sp. Nov., a chemolithoheterotroph isolated from sulfide-and organic-rich coastal waters off Peru. Applied and Environmental Microbiology. 2019;85: e01344–19. doi: 10.1128/AEM.01344-19 31585991 PMC6881792

[29] Ohde T. Coastal Sulfur Plumes off Peru During El Niño, La Niña, and Neutral Phases. Geophysical Research Letters. 2018;45: 7075–7083. doi: 10.1029/2018GL077618

[30] Ohde T, Siegel H, Reißmann J, Gerth M. Identification and investigation of sulphur plumes along the Namibian coast using the MERIS sensor. Continental Shelf Research. 2007;27: 744–756. doi: 10.1016/j.csr.2006.11.016

[31] Weeks SJ, Currie B, Bakun A, Peard KR. Hydrogen sulphide eruptions in the Atlantic Ocean off southern Africa: Implications of a new view based on SeaWiFS satellite imagery. Deep-Sea Research Part I: Oceanographic Research Papers. 2004;51: 153–172. doi: 10.1016/j.dsr.2003.10.004

[32] Sommer S, Gier J, Treude T, Lomnitz U, Dengler M, Cardich J, et al. Depletion of oxygen, nitrate and nitrite in the Peruvian oxygen minimum zone cause an imbalance of benthic nitrogen fluxes. Deep-Sea Research Part I: Oceanographic Research Papers. 2016;112: 113–122. doi: 10.1016/j.dsr.2016.03.001

[33] Ohde T, Dadou I. Seasonal and annual variability of coastal sulphur plumes in the northern Benguela upwelling system. PloS one. 2018;13: e0192140. doi: 10.1371/journal.pone.0192140 29420587 PMC5805238

[34] Dale AW, Graco M, Wallmann K. Strong and dynamic benthic-pelagic coupling and feedbacks in a coastal upwelling system (Peruvian shelf). Frontiers in Marine Science. 2017;4: 29.

[35] Naqvi SWA, Naik H, Jayakumar D, Shailaja M, Narvekar P. Seasonal oxygen deficiency over the western continental shelf of India. Past and present water column anoxia. Springer; 2006. Pp. 195–224.

[36] Thomsen S, Kanzow T, Krahmann G, Greatbatch RJ, Dengler M, Lavik G. The formation of a subsurface anticyclonic eddy in the P eru-C hile U ndercurrent and its impact on the near-coastal salinity, oxygen, and nutrient distributions. Journal of Geophysical Research: Oceans. 2016;121: 476–501.

[37] Thamdrup B, Dalsgaard T, Revsbech NP. Widespread functional anoxia in the oxygen minimum zone of the Eastern South Pacific. Deep Sea Research Part I: Oceanographic Research Papers. 2012;65: 36–45.

[38] Dale AW, Sommer S, Lomnitz U, Montes I, Treude T, Liebetrau V, et al. Organic carbon production, mineralisation and preservation on the Peruvian margin. Biogeosciences. 2015;12: 1537–1559.

[39] Gutiérrez D, Enríquez E, Purca S, Quipúzcoa L, Marquina R, Flores G, et al. Oxygenation episodes on the continental shelf of central Peru: Remote forcing and benthic ecosystem response. Progress in Oceanography. 2008;79: 177–189. doi: 10.1016/j.pocean.2008.10.025

[40] Chaigneau A, Dominguez N, Eldin G, Vasquez L, Flores R, Grados C, et al. Near-coastal circulation in the Northern Humboldt Current System from shipboard ADCP data. Journal of Geophysical Research: Oceans. 2013;118: 5251–5266.

[41] Chaigneau A, Eldin G, Dewitte B. Eddy activity in the four major upwelling systems from satellite altimetry (1992–2007). Progress in Oceanography. 2009;83: 117–123.

[42] Chaigneau A, Gizolme A, Grados C. Mesoscale eddies off Peru in altimeter records: Identification algorithms and eddy spatio-temporal patterns. Progress in Oceanography. 2008; 79: 106–119.

[43] Chauca Z. Caracterización de los eventos de aguas blancas frente a pisco y chincha (entre los 13°-15° s). 2018; 204.

[44] Siegel H, Ohde T, Gerth M, Lavik G, Leipe T. Identification of coccolithophore blooms in the SE Atlantic Ocean off Namibia by satellites and in-situ methods. Continental Shelf Research. 2007;27: 258–274.

[45] Wright JJ, Konwar KM, Hallam SJ. Microbial ecology of expanding oxygen minimum zones. Nature Reviews Microbiology. 2012;10: 381–394. doi: 10.1038/nrmicro2778 22580367

[46] Merma-Mora L. Foraminíferos bentónicos asociados a condiciones de hipoxia costera y bajo pH en la Bahía de Paracas. M.Sc. Thesis, Universidad Peruana Cayetano Heredia. 2016. https://hdl.handle.net/20.500.12866/240.

[47] Aguirre-Velarde A, Thouzeau G, Jean F, Mendo J, Cueto-Vega R, Kawazo-Delgado M, et al. Chronic and severe hypoxic conditions in Paracas Bay, Pisco, Peru: Consequences on scallop growth, reproduction, and survival. Aquaculture. 2019;512: 734259. doi: 10.1016/j.aquaculture.2019.734259

[48] Strickland JDH, Parsons TR. A practical handbook of seawater analysis. 1972.

[49] Seeberg-Elverfeldt J, Schlüter M, Feseker T, Kölling M. Rhizon sampling of porewaters near the sediment-water interface of aquatic systems. Limnology and oceanography: Methods. 2005;3: 361–371.

[50] Fonseca A, Ishoey T, Espinoza C, Perez-Pantoja D, Manghisi A, Morabito M, et al. Genomic features of “Candidatus Venteria ishoeyi”, a new sulfur-oxidizing macrobacterium from the Humboldt Sulfuretum off Chile. Plos one. 2017;12: e0188371. doi: 10.1371/journal.pone.0188371 29236755 PMC5728499

[51] Fossing H, Gallardo VA, Jørgensen BB, Hüttel M, Nielsen LP, Schulz H, et al. Concentration and transport of nitrate by the mat-forming sulphur bacterium Thioploca. Nature. 1995;374: 713–715.

[52] Cline JD. Spectrophotometric determination of hydrogen sulfide in natural waters 1. Limnology and Oceanography. 1969;14: 454–458.

[53] Schulz H, Brinkhoff T, Ferdelman TG, Mariné MH, Teske A, Jørgensen BB. Dense populations of a giant sulfur bacterium in Namibian shelf sediments. Science. 1999;284: 493–495. doi: 10.1126/science.284.5413.493 10205058

[54] Bohlen L, Dale AW, Sommer S, Mosch T, Hensen C, Noffke A, et al. Benthic nitrogen cycling traversing the Peruvian oxygen minimum zone. Geochimica et Cosmochimica Acta. 2011;75: 6094–6111.

[55] Berner RA, Berner RA. Principles of chemical sedimentology. McGraw-Hill New York; 1971.

[56] Boudreau BP. Diagenetic models and their implementation. Springer Berlin; 1997.

[57] Yuan-Hui L, Gregory S. Diffusion of ions in sea water and in deep-sea sediments. Geochimica et cosmochimica acta. 1974;38: 703–714.

[58] Legendre P, Borcard D, Blanchet G, Dray S. PCNM: PCNM spatial eigenfunction and principal coordinate analyses. 2011.

[59] Team RC, others. R: A language and environment for statistical computing; 2018. 2018.

[60] Gutiérrez D, Bouloubassi I, Sifeddine A, Purca S, Goubanova K, Graco M, et al. Coastal cooling and increased productivity in the main upwelling zone off Peru since the mid-twentieth century. Geophysical Research Letters. 2011;38: 1–6. doi: 10.1029/2010GL046324

[61] Messié M, Chavez FP. Seasonal regulation of primary production in eastern boundary upwelling systems. Progress in Oceanography. 2015;134: 1–18.

[62] Pitcher GC, Aguirre-Velarde A, Breitburg D, Cardich J, Carstensen J, Conley DJ, et al. System controls of coastal and open ocean oxygen depletion. Progress in Oceanography. 2021; 102613.

[63] Igarza M, Boussafir M, Graco M, Sifeddine A, Valdés J, Gutiérrez D. Latitudinal variability of preserved sedimentary organic matter along the Peruvian continental margin as inferred from petrographic and geochemical properties. Marine Chemistry. 2021;235: 104004.

[64] Suess E, Kulm L, Killingley J. Coastal upwelling and a history of organic-rich mudstone deposition off Peru. Geological Society, London, Special Publications. 1987;26: 181–197.

[65] Böning P, Brumsack HJ, Böttcher ME, Schnetger B, Kriete C, Kallmeyer J, et al. Geochemistry of Peruvian near-surface sediments. Geochimica et Cosmochimica Acta. 2004;68: 4429–4451. doi: 10.1016/j.gca.2004.04.027

[66] Lomovasky BJ, Firstater FN, Salazar AG, Mendo J, Iribarne OO. Macro benthic community assemblage before and after the 2007 tsunami and earthquake at Paracas Bay, Peru. Journal of Sea Research. 2011;65: 205–212. doi: 10.1016/j.seares.2010.10.002

[67] Brocławik O, Łukawska-Matuszewska K, Brodecka-Goluch A, Bolałek J. Impact of methane occurrence on iron speciation in the sediments of the Gdansk Basin (Southern Baltic Sea). Science of The Total Environment. 2020;721: 137718. doi: 10.1016/j.scitotenv.2020.137718 32179345

[68] Echevin V, Aumont O, Ledesma J, Flores G. The seasonal cycle of surface chlorophyll in the Peruvian upwelling system: A modelling study. Progress in Oceanography. 2008;79: 167–176. doi: 10.1016/j.pocean.2008.10.026

[69] Maltby J, Sommer S, Dale AW, Treude T. Microbial methanogenesis in the sulfate-reducing zone of surface sediments traversing the Peruvian margin. Biogeosciences. 2016;13: 283–299. doi: 10.5194/bg-13-283-2016

[70] Vargas CA, Cantarero SI, Sepúlveda J, Galán A, De Pol-Holz R, Walker B, et al. A source of isotopically light organic carbon in a low-pH anoxic marine zone. Nature communications. 2021;12: 1–11. 10.1038/s41467-021-21871-4 PMC7952585 33707435

[71] Brüchert V, Currie B, Peard KR, Lass U, Endler R, Dübecke A, et al. Biogeochemical and physical control on shelf anoxia and water column hydrogen sulphide in The Benguel a coastal upwelling system off Namibia. Past and present water column anoxia. Springer; 2006. Pp. 161–193.

[72] Elrod VA, Berelson WM, Coale KH, Johnson KS. The flux of iron from continental shelf sediments: A missing source for global budgets. Geophysical Research Letters. 2004;31.

[73] Croot PL, Heller MI, Wuttig K. Redox Processes Impacting the Flux of Iron(II) from Shelf Sediments to the OMZ along the Peruvian Shelf. ACS Earth and Space Chemistry. 2019;3: 537–549. doi: 10.1021/acsearthspacechem.8b00203

