# Supplementary figures and images for: Attenuation of wind intensities exacerbates anoxic conditions leading to sulfur plume development off the coast of Peru
Source: PLoS One. 2023 Aug 30;18(8):e0287914. doi: 10.1371/journal.pone.0287914 (PMC10468053; doi:10.1371/journal.pone.0287914)

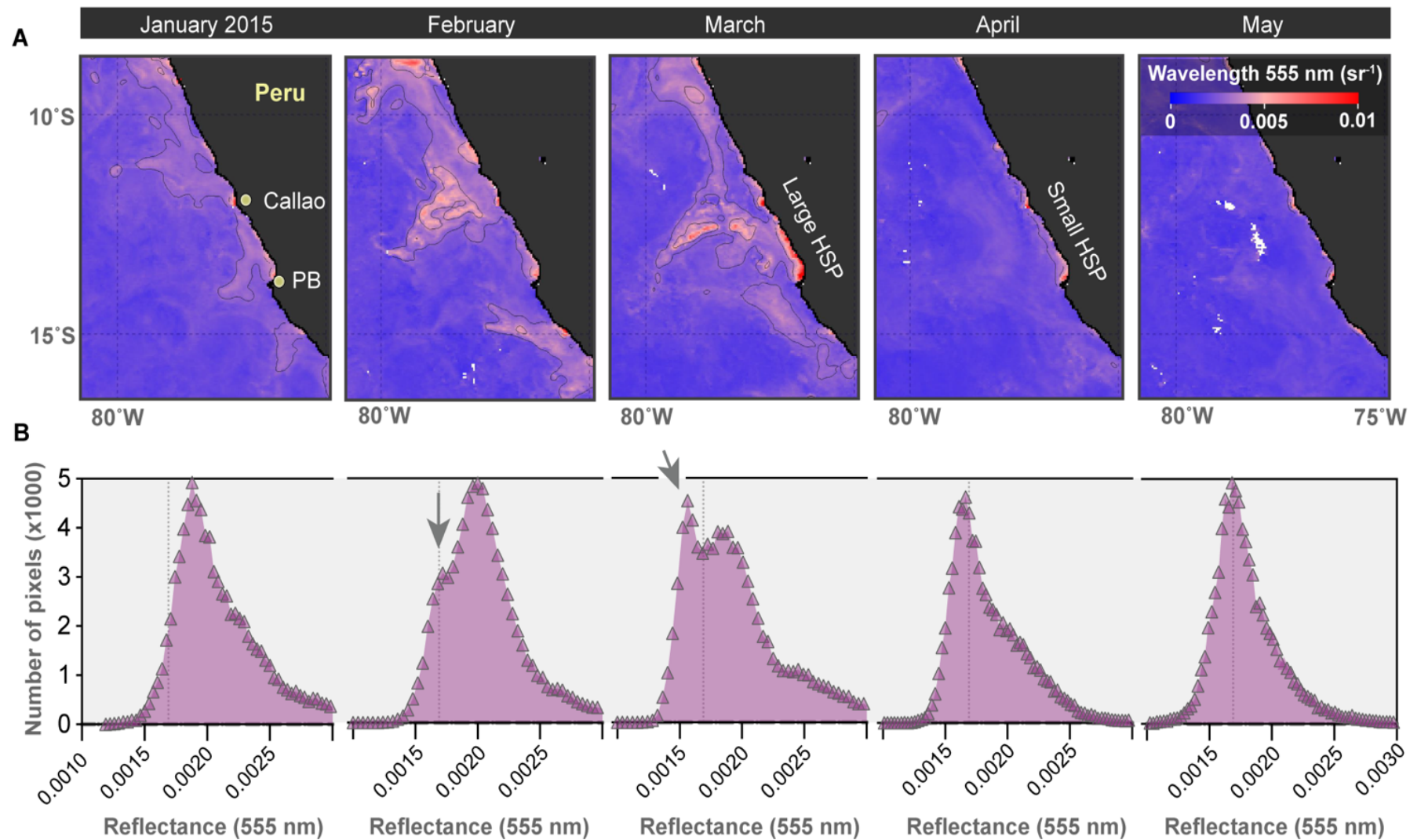

Supplement: S1 Fig — (A) MODIS images of the elemental sulfur plume as detected by the water reflectance wavelength at 555 nm (optimal band for ESP detection). (B) Pixel histograms collected for the water reflectance wavelength at 555 nm. The ESP event(s) detected in February and March were denoted by the presence of high intensity sulfur-containing pixels (indicated by the grey arrows), resulting in a bimodal histogram profile. The March bimodal histogram is especially distinct from the May monomodal histogram profile, which represents a non-ESP scenario (i.e., post El Nino). As discussed in the methods, we performed a background excess calculation, in that, the binned pixels (at 555 nm) associated with a non-ESP month (May) were subtracted from the binned sulfur-containing pixels of the March 2015 period (ESP scenario). Given the known area of a pixel, we deduced that the March ESP was an estimated 16,963 km2. (PDF) [file pone.0287914.s001.pdf]

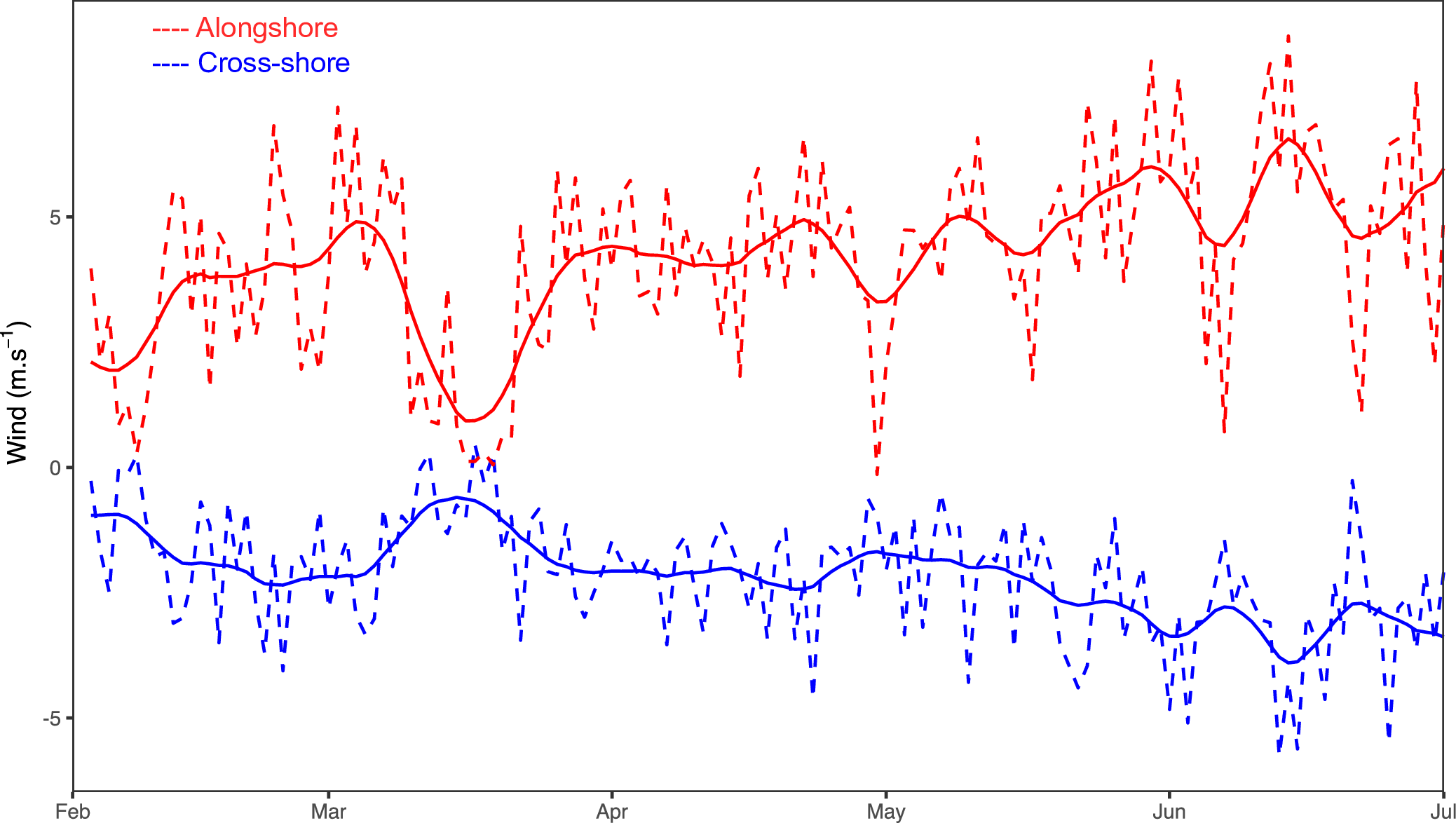

Supplement: S2 Fig — The wind velocity data was acquired by Advanced Scatterometer (ASCAT) aboard Metop-A and Metop-B satellites, during the austral summer-autumn period in 2015, off the coast of Peru (see also Fig 1A). (TIF) [file pone.0287914.s002.tif]

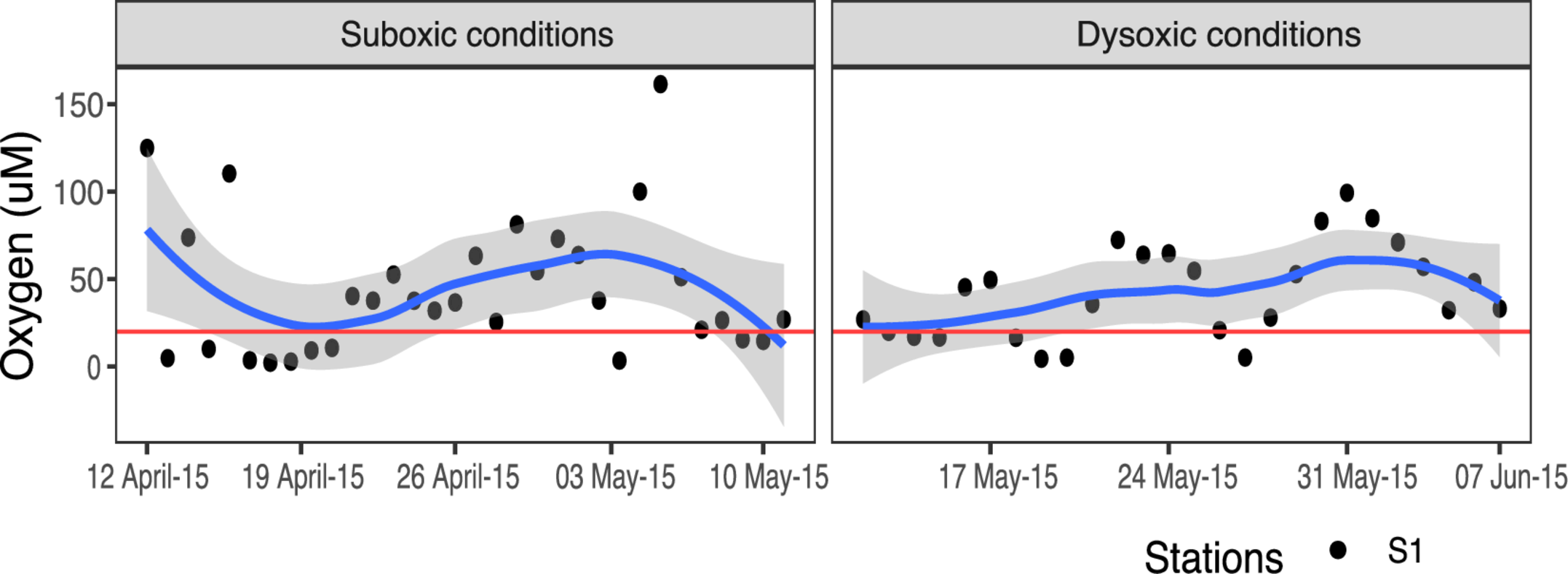

Supplement: S3 Fig — Synoptic time series images were taken during the austral summer-autumn period in 2015. In both panels (A, B), the blue line denotes the smoothed daily mean, while the grey shading illustrates the confidence interval. The red line indicates the dissolved oxygen threshold for suboxic (< 20 μM) and dysoxic (20–90 μM) conditions, as previously defined according to Wright [42]. (TIF) [file pone.0287914.s003.tif]

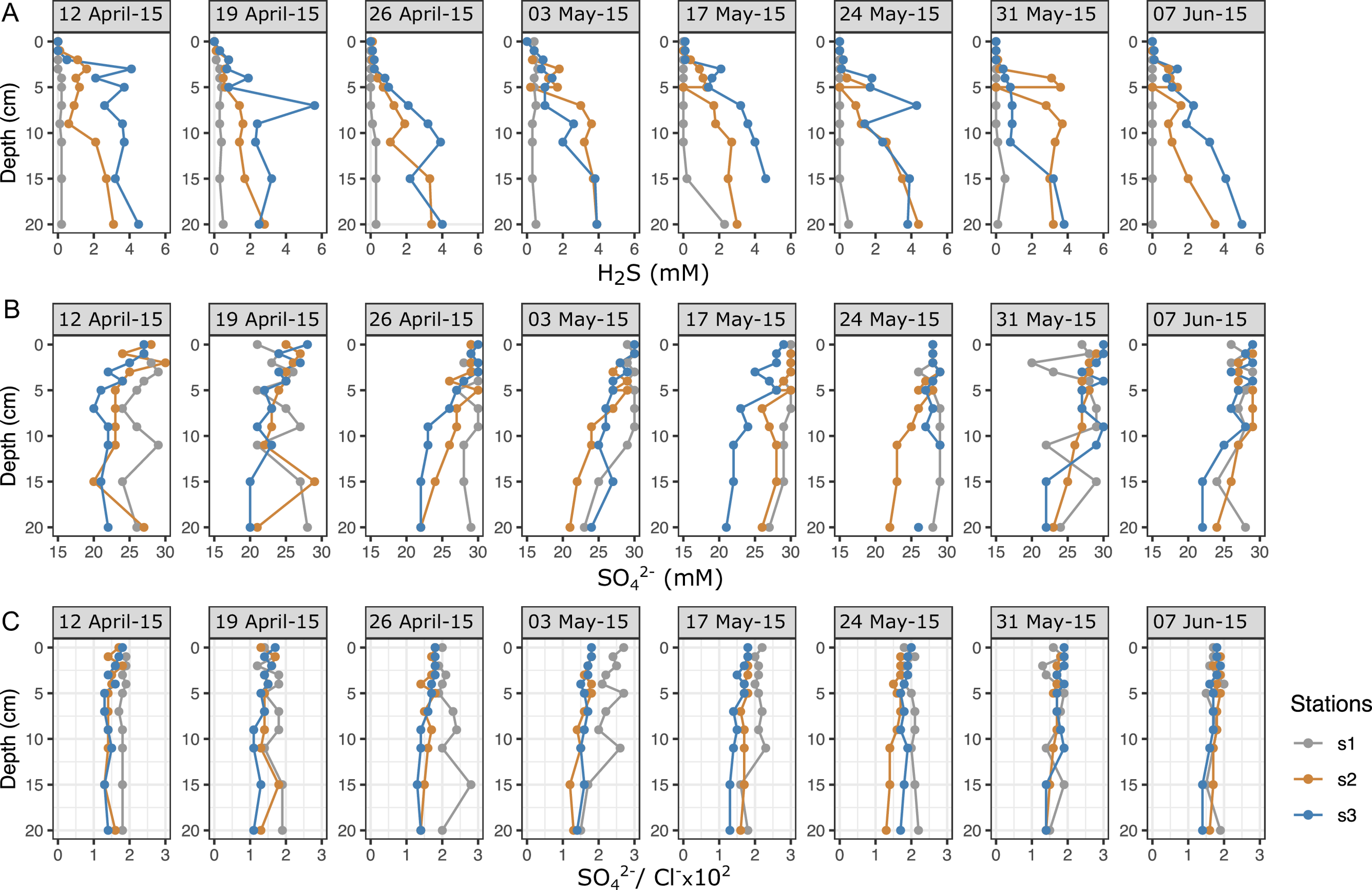

Supplement: S4 Fig — Pore water concentrations of (A) sulfide, (B) sulfate, and (C) sulfate/chloride ratio are shown. Note that the arrival of the El Nino event (i.e., re-oxygenation of the water column) occurred from May 17 onward. Prior to the El Nino event, the water column was largely void of both oxygen and nitrate, see also Fig 3A–3C. (TIF) [file pone.0287914.s004.tif]

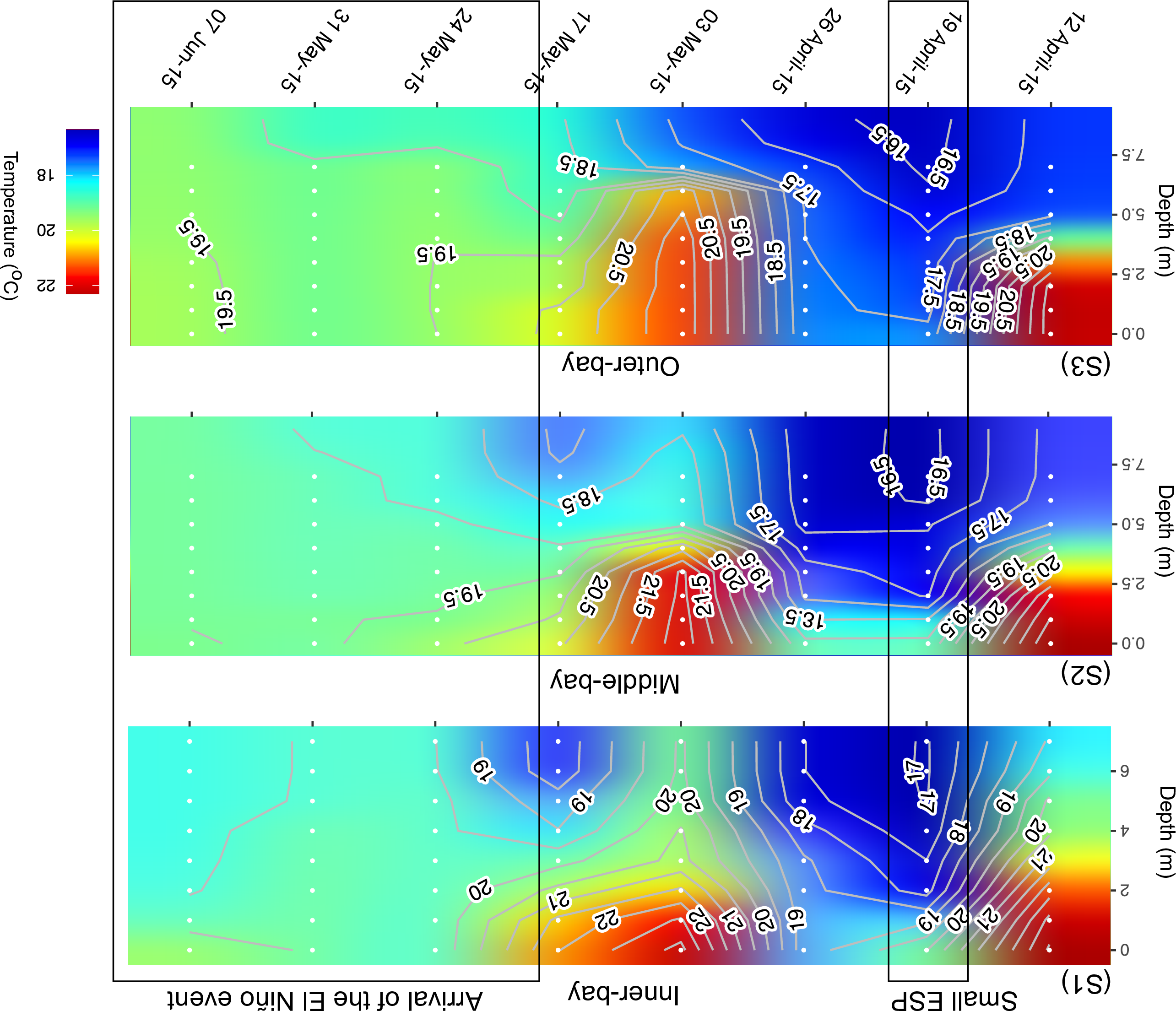

Supplement: S5 Fig — Temperature (°C) measured in situ, linearly interpolated to 1 m depth intervals at the three stations (S1, S2, & S3). The black box shows the arrival of the 2015 El Niño event. (TIF) [file pone.0287914.s005.tif]
